# *GTR1* Affects Nitrogen Consumption and TORC1 Activity in *Saccharomyces cerevisiae* Under Fermentation Conditions

**DOI:** 10.3389/fgene.2020.00519

**Published:** 2020-05-25

**Authors:** Jennifer Molinet, Francisco Salinas, José Manuel Guillamón, Claudio Martínez

**Affiliations:** ^1^Departamento de Ciencia y Tecnología de los Alimentos, Universidad de Santiago de Chile (USACH), Santiago, Chile; ^2^Millennium Institute for Integrative Biology (iBio), Santiago, Chile; ^3^Centro de Estudios en Ciencia y Tecnología de Alimentos (CECTA), Universidad de Santiago de Chile (USACH), Santiago, Chile; ^4^Instituto de Bioquímica y Microbiología, Facultad de Ciencias, Universidad Austral de Chile, Valdivia, Chile; ^5^Departamento de Biotecnología de los Alimentos, Instituto de Agroquímica y Tecnología de los Alimentos (CSIC), Valencia, Spain

**Keywords:** *Saccharomyces cerevisiae*, wine fermentation, *GTR1* gene, TORC1 pathway, allelic diversity

## Abstract

The TORC1 pathway coordinates cell growth in response to nitrogen availability present in the medium, regulating genes related to nitrogen transport and metabolism. Therefore, the adaptation of *Saccharomyces cerevisiae* to changes in nitrogen availability implies variations in the activity of this signaling pathway. In this sense, variations in nitrogen detection and signaling pathway are one of the main causes of differences in nitrogen assimilation during alcoholic fermentation. Previously, we demonstrated that allelic variants in the *GTR1* gene underlying differences in ammonium and amino acids consumption between Wine/European (WE) and West African (WA) strains impact the expression of nitrogen transporters. The *GTR1* gene encodes a GTPase that participates in the EGO complex responsible for TORC1 activation in response to amino acids availability. In this work, we assessed the role of the *GTR1* gene on nitrogen consumption under fermentation conditions, using a high sugar concentration medium with nitrogen limitation and in the context of the WE and WA genetic backgrounds. The *gtr1Δ* mutant presented a reduced TORC1 activity and increased expression levels of nitrogen transporters, which in turn favored ammonium consumption, but decreased amino acid assimilation. Furthermore, to identify the SNPs responsible for differences in nitrogen consumption during alcoholic fermentation, we studied the polymorphisms present in the *GTR1* gene. We carried out swapping experiments for the promoter and coding regions of *GTR1* between the WE and WA strains. We observed that polymorphisms in the coding region of the WA *GTR1* gene are relevant for TORC1 activity. Altogether, our results highlight the role of the *GTR1* gene on nitrogen consumption in *S. cerevisiae* under fermentation conditions.

## Introduction

Nitrogen is one of the most important nutrients present in grape must affecting various outcomes of alcoholic fermentation (Tesnière et al., 2015). The main microorganism responsible for this process is *Saccharomyces cerevisiae* (Pretorius, 2000), which responds to changes in nitrogen availability by adjusting its metabolism to new environmental conditions. The adaptation of *S. cerevisiae* strains to nitrogen availability involves changes in signaling pathway and gene expression (Rossignol et al., 2003; Brice et al., 2014b, 2018; Tesnière et al., 2015). However, the responses of *S. cerevisiae* to changes in nitrogen availability have been poorly studied under conditions that mimic industrial fermentation, where yeast has to face a stressful environment with high concentration of sugar (20% of glucose and fructose) and ethanol, low pH (pH 3.0–4.0), anaerobiosis (low O_2_) and limited availability of nitrogen (Pretorius, 2000; Kessi-Pérez et al., 2020).

Grape musts contain a wide variety of nitrogen sources, however, yeast have a hierarchical preference for their consumption (Magasanik and Kaiser, 2002) favoring ammonium and amino acids (Bell and Henschke, 2005). In this sense, yeasts coordinate their protein synthesis and growth rate according to the quantity and quality of available nitrogen (Broach, 2012). Under fermentation conditions using a culture medium that mimics industrial fermentation, yeasts initially consume nitrogen sources whose permeases are regulated by the Ssy1-Ptr3-Ssy5 system (SPS), followed by nitrogen sources whose transporters are regulated by the Nitrogen Catabolite Repression (NCR) system (Crépin et al., 2012). Once the nitrogen sources are assimilated, most are incorporated into the Nitrogen Central Metabolism, transformed into glutamate or glutamine, and stored as amino acid pools in the cytoplasm, while positively charged amino acids such as arginine are stored in the vacuole (Crépin et al., 2014, 2017; Gutiérrez et al., 2016). The intracellular pools are used when the extracellular nitrogen is depleted, incorporating them in *de novo* amino acid synthesis or directly into proteins to initiate cell growth (Gutiérrez et al., 2016; Crépin et al., 2017). However, *S. cerevisiae* strains present different nitrogen consumption profiles (Contreras et al., 2012; Gutiérrez et al., 2012; Brice et al., 2014b, 2018; Jara et al., 2014; Cubillos et al., 2017), where variations in nitrogen metabolism and signaling are largely responsible for these phenotypic differences (Brice et al., 2014a; Cubillos et al., 2017; Molinet et al., 2019).

Recently, we demonstrated that a large diversity exists within the TORC1 pathway and allelic variants from this pathway significantly impact nitrogen metabolism, influencing the nitrogen consumption differences between strains representative of four clean lineages described in *S. cerevisiae* (Wine/European WE, West African WA, North American NA, and Sake SA) (Molinet et al., 2019). Furthermore, during alcoholic fermentation, TORC1 plays a key role in controlling the expression of genes related to nitrogen utilization (Rossignol et al., 2003), and on the fermentative capacity in nitrogen starved cells (Brice et al., 2014a). Similarly, Kessi-Pérez et al. (2019b) demonstrated a large phenotypic variation in TORC1 activity between the same four strains, with the greatest TORC1 activity observed in the SA strain and the lowest in the WE strain. Interestingly, the SA strain has a preference for amino acids consumption, while the WE strain presents a preference for ammonium (Cubillos et al., 2017). Thus, variations in the TORC1 activity imply differential activation of the downstream pathways such as NCR and SPS systems, which ultimately affect nitrogen consumption.

The TORC1 pathway coordinates cell growth in response to nutrient availability (De Virgilio and Loewith, 2006; Broach, 2012; Conrad et al., 2014; Zhang et al., 2018), predominantly detecting the quantity and quality of available nitrogen sources (Broach, 2012). TOCR1 activity is inhibited under nitrogen depletion conditions, whereas its activity increases upon nitrogen upshift. Its main downstream targets are the protein kinase Sch9p and the Tap42-PP2A complex, positively regulating ribosomal biogenesis and translation, and inhibiting the stress response (Conrad et al., 2014). TORC1 is regulated by the EGO complex (EGOC) composed of four proteins: Ego1p, Ego3p, Grt1p, and Gtr2p. This complex regulates TORC1 interacting physically with its subunits. Gtr1p and Gtr2p are Ras-family GTPases. When Gtr1p binds to GTP and Gtr2p to GDP it forms a heterodimeric complex resulting in the activation of TORC1 by EGOC (Hatakeyama and De Virgilio, 2016). Therefore, the localization of EGOC in the vacuole outer membrane, next to TORC1, allows the detection of amino acid levels and regulates TORC1 activity. Hence, this signal may involve mobilization of amino acids from their stores in the intracellular pools or vacuole (Broach, 2012).

The TORC1 pathway activity has been poorly studied under fermentation conditions, with the majority of studies done at the transcriptional level (Rossignol et al., 2003; Brice et al., 2014b; Walker et al., 2014; Tesnière et al., 2015). Previously, we identified seven genes of the TORC1 pathway (*GTR1, SAP185, SIT4, EAP1, NPR1, EAP1*, and *TOR2*) whose allelic diversity affects nitrogen consumption during alcoholic fermentation. We observed that *GTR1* allelic variants affect the consumption of amino acids and ammonium, where the Wine/European allele (WE) of this gene presented a preference for ammonium consumption, whereas the West African allele (WA) showed a preference for amino acids, similarly to that reported by Cubillos et al. (2017). Furthermore, the presence of the WE allele resulted in a higher expression level of ammonium permeases (*MEP1, MEP2* and *MEP3*) in line with higher ammonium consumption (Molinet et al., 2019). Therefore, considering that the regulation of TORC1 activity could be relevant to nitrogen consumption, we decided to further study the role of the *GTR1* gene on this phenotype.

In this work, we assessed the role of the *GTR1* gene on nitrogen consumption under fermentation conditions, using a media with high sugar concentration and nitrogen limitation, in the WE and WA genetic backgrounds. We evaluated nitrogen consumption in the *gtr1Δ* mutant and in different strains with swapped regulatory and coding regions for *GTR1*. The results showed a reduced TORC1 activation and higher expression levels of nitrogen transporters in the *gtr1Δ* mutant, favoring ammonium consumption but decreasing amino acid assimilation. Finally, a SNP in the coding region of the *GTR1* gene is relevant for TORC1 activity in the WA strain. Altogether, our results indicate that *GTR1* regulates nitrogen consumption under fermentation conditions in *S. cerevisiae*.

## Materials and Methods

### Strains and Culture Media

The haploid strains used in this work correspond to DBVPG6765 (WE, Wine/European, *Mat α, ho::HygMX, ura3::KanMX*) and DBVPG6044 (WA, West African, *Mat α, ho::HygMX, ura3::KanMX*), previously described by Cubillos et al. (2009). All the strains were maintained on YPD solid media (1% yeast extract, 2% peptone, 2% glucose, 2% agar).

Synthetic must (SM) used in fermentations has been previously described (Bely et al., 1990; Rossignol et al., 2003; Martínez et al., 2014) and contains a mixture of sugar (125 g L^–1^ of glucose and 125 g L^–1^ of fructose), mineral salts (750 mg L^–1^ KH_2_PO_4_, 500 mg L^–1^ K_2_SO_4_, 250 mg L^–1^ MgSO_4_⋅7H_2_O, 155 mg L^–1^ CaCl_2_⋅2H_2_O, 200 mg L^–1^ NaCl, 4 mg L^–1^ MnSO_4_⋅H_2_O, 4 mg L^–1^ ZnSO_4_⋅7H_2_O, 1 mg L^–1^ CUSO_4_⋅5H_2_O, 1 mg L^–1^ KI, 0.4 mg L^–1^ CoCl_2_⋅6H_2_O, 1 mg L^–1^ H_3_BO_3_ and 1 mg L^–1^ NaMoO_4_⋅2H_2_O), vitamins (20 mg L^–1^ myo-inositol, 2 mg L^–1^ nicotinic acid, 1.5 mg L^–1^ calcium panthothenate, 0.25 mg L^–1^ thiamine HCl, 0.25 mg L^–1^ pyridoxine HCl and 0.003 mg L^–1^ biotin) and anaerobic factors (15 mg L^–1^ ergosterol, 5 mg L^–1^ sodium oleate) in Tween 80/ethanol solution (50/50 v/v). SM medium was adjusted to pH 3.3 with HCl and filtered. The final concentration of yeast assimilable nitrogen was 300 mg L^–1^ to SM300 and 140 mg L^–1^ to SM140 medium. The nitrogen concentration in SM300 corresponded to 120 mgN L^–1^ of ammonium and 180 mgN L^–1^ of amino acids mixture (612.6 mg L^–1^ L-proline, 503.5 mg L^–1^ L-glutamine, 503.5 mg L^–1^ L-arginine monohydrochloride, 179.3 mg L^–1^ L-tryptophan, 145.3 mg L^–1^ L-alanine, 120.4 mg L^–1^ L-glutamic acid, 78.5 mg L^–1^ L-serine, 75.92 mg L^–1^ L-threonine, 48.4 mg L^–1^ L-leucine, 44.5 mg L^–1^ L-aspartic acid, 44.5 mg L^–1^ L-valine, 37.9 mg L^–1^ L-phenylalanine, 32–7 mg L^–1^ L-isoleucine, 50.0 mg L^–1^ L-histidine monohydrochloride monohydrate, 31.4 mg L^–1^ L-methionine, 18.3 mg L^–1^ L-tyrosine, 18.3 mg L^–1^ L-glycine, 17.0 mg L^–1^ L-lysine monohydrochloride, and 13.1 mg L^–1^ L-cysteine). SM140-ammonium medium corresponds to synthetic must previously described, but with a final concentration of 140 mgN L^–1^ of ammonium. In the same way, SM140-amino acids medium presented a final concentration of 140 mgN L^–1^ of amino acids mixture (612.6 mg L^–1^ L-proline, 503.5 mg L^–1^ L-glutamine, 503.5 mg L^–1^ L-arginine monohydrochloride, 179.3 mg L^–1^ L-tryptophan, 145.3 mg L^–1^ L-alanine, 120.4 mg L^–1^ L-glutamic acid, 78.5 mg L^–1^ L-serine, 75.92 mg L^–1^ L-threonine, 48.4 mg L^–1^ L-leucine, 44.5 mg L^–1^ L-aspartic acid, 44.5 mg L^–1^ L-valine, 37.9 mg L^–1^ L-phenylalanine, 32–7 mg L^–1^ L-isoleucine, 50.0 mg L^–1^ L-histidine monohydrochloride monohydrate, 31.4 mg L^–1^ L-methionine, 18.3 mg L^–1^ L-tyrosine, 18.3 mg L^–1^ L-glycine, 17.0 mg L^–1^ L-lysine monohydrochloride, and 13.1 mg L^–1^ L-cysteine).

### Generation of Null Mutants and Strains by Allele Swapping

The null mutants were previously described (Kessi-Pérez et al., 2019b; Molinet et al., 2019). The strains with the swapping promoter and coding regions of *GTR1* were obtained by *in vivo* assembly recombinational cloning (Oldenburg et al., 1997; Salinas et al., 2016). Briefly, the constructs containing the promoter, ORF and hygromycin cassette (hphMx) were designed *in silico* considering the same transcriptional orientation to avoid convergent transcription using the Geneious software 8.1.8 (Biomatters, New Zealand). Then, the promoter, ORF and hphMX were PCR amplified using Phusion High-Fidelity DNA polymerase (Thermo Fisher Scientific, United States). The yeast strain BY4741 (*MATa, his3Δ1, leu2Δ0, LYS2, met15Δ0, ura3Δ0*) was transformed using PCR products with 50–70 bp of overlap between them, and the linear plasmid pRS426. The circular plasmids were then recovered from the yeast and transferred to an *E. coli* DH5α strain. Plasmids from three positive colonies for each construct were purified and sequenced using the standard Sanger sequencing service (Macrogen, South Korea). The sequences were analyzed using the SGRP (Saccharomyces Genome Resequencing Project) BLAST^1^ server and MUSCLE^2^. The complete constructs were PCR amplified using Phusion High-Fidelity DNA polymerase (Thermo Fisher Scientific, United States), using primers with 70 bp of homology with the target locus (Supplementary Table S1), and used to transform the WE and WA strains. Five different colonies for each construct were confirmed by standard yeast colony PCR.

### Fermentation in Synthetic Must

Fermentations in synthetic must (SM300) (Bely et al., 1990; Rossignol et al., 2003) were carried out in nine biological replicates as previously described (Cubillos et al., 2017; Molinet et al., 2019). The pre-cultures were grown in SM300 for 24 h at 25°C without agitation. Then, 12 mL of SM300 were inoculated with 1 × 10^6^ cells mL^–1^ and incubated at 25°C for 20 days without agitation. Fermentations were monitored weighing the tubes daily and determining weight loss over time. The kinetic parameters (the maximal CO_2_ production rate V_max_, V_50_/V_max_ ratio and efficiency) (Marullo et al., 2006) were calculated from the CO_2_ loss curves previously fitted with a sigmoid non-linear regression (Martinez et al., 2013).

### Determination of Nitrogen Consumption

Nitrogen consumption was determined in three independent biological replicates as previously described (Cubillos et al., 2017; Molinet et al., 2019). Briefly, supernatants from fermentations were collected by centrifugation at 9000xg for 10 min and the concentration of ammonium and the 19 amino acids present in the must were determined by derivatization with diethyl ethoxymethylenemalonate (DEEMM) (Gómez-Alonso et al., 2007). Subsequently, they were separated by HPLC using a Bio-Rad HPX-87H column in a Shimadzu Prominence HPLC equipment (Shimadzu, United States) (Nissen et al., 1997). This analysis was carried out on the sixth day of fermentation where most of the nitrogen consumption differences can be observed (Martinez et al., 2013; Jara et al., 2014). The uptake of each nitrogen source was estimated as the difference between the initial and final concentration before and after fermentation, respectively (Jara et al., 2014; Cubillos et al., 2017).

### Determination of Growth Variables

Growth variables were determined from micro-cultivation experiments in SM300, SM140- ammonium, and SM140-amino acids media at 30°C for 48 h. The pre-cultures were grown in synthetic must medium at 30°C for 24 h and used to inoculate a 96-well plate with a final volume of 200 μL at an initial OD_600 nm_ of 0.1. The growth curves were monitored by measuring the OD_600 nm_ every 20 min in a Tecan Sunrise absorbance microplate reader (Tecan Group Ltd., Switzerland). The experiments were all carried out in three biological replicates. Lag phase, growth efficiency (Δ OD_600 nm_) and the maximum specific growth rate (μ_max_) were determined as previously described (Warringer and Blomberg, 2003; Ibstedt et al., 2015). For this, the parameters were calculated following curve fitting (OD values were transformed to ln) utilizing the Gompertz function (Zwietering et al., 1990) using the R software version 3.3.2.

### RNA Extraction and qPCR Assay

Gene expression analysis was performed by qPCR from fermentations in SM300 medium as previously described (Molinet et al., 2019). Briefly, cells at 6 h of fermentation were collected by centrifugation and treated with 10 units of Zymolyase (Seikagaku Corporation, Japan) for 30 min at 37°C. RNA was extracted using the E.Z.N.A Total RNA Kit I (OMEGA) according to manufacturers’ instructions. Then, genomic DNA traces were removed by treating samples with DNase I (Promega) and total RNA was recovered using the GeneJET RNA Cleanup and Concentration Micro Kit (Thermo Fisher Scientific). Concentrations of the purified RNA were determined with a UV-Vis spectrophotometer EPOCH equipment (BioTeK Instruments Inc., United States) and verified on 1.5% agarose gels. The RNA extractions were performed in two biological replicates.

cDNA was synthesized using one unit of M-MLV Reverse transcriptase (Promega), 0.4 μg of Oligo (dT)_15_ primer and 0.8 μg of RNA in a final volume of 25 μL according to manufacturers’ instructions. cDNA samples obtained were quantified using a UV-Vis spectrophotometer EPOCH equipment (BioTeK Instruments Inc., United States). The qPCR reactions were carried out using HOT FIREPol EvaGreen qPCR Mix Plus (Solis BioDyne) in a final volume of 20 μL, containing 0.25 μM of each primer and 1 μL of the cDNA previously synthesized. The qPCR reactions were carried out in three technical replicates per biological replicate using a Step One Plus Real-Time PCR System (Applied Biosystems, United States) under the following conditions: 95°C for 15 min and 40 cycles at 95°C for 15 s and 55°C for 15 s. The genes and primers used are listed in Supplementary Table S1. The mathematical method described by Pfaffl (2001) was used to quantify the relative expression of each gene. Expression levels were normalized with three housekeeping genes *ACT1*, *UBC6* and *RPN2* (Teste et al., 2009) according to Vandesompele et al. (2002). The ΔCt were analyzed using the nonparametric Mood test (Yuan et al., 2006).

### TORC1 Pathway Activation in Micro-Culture Conditions

The TORC1 pathway activation was monitored using an indirect method described by Kessi-Pérez et al. (2019b), employing the expression of the *RPL26A* gene as an indirect readout for the activity status of TORC1. For this, we swapped the *RPL26A* ORF with the *Luc-URA3* reporter construct and measured the luminescence produced by yeast cells in micro-culture conditions. The strains carrying the *Luc-URA3* reporter construct were generated as described by Kessi-Pérez et al. (2019b). The pre-cultures were grown in synthetic must medium at 30°C for 24 h and used to inoculate a 96-well plate with a final volume of 300 μL supplemented with luciferin (1 mM) at an initial OD_600 nm_ of 0.1 and growth at 30°C. Luminescence was measured up to 24 h using 30 min intervals at 30°C in a Synergy HTX plate reader (BioTek, United States). All the experiments were performed in three biological replicates. From the luminescence curves three parameters were extracted: maximum luminescence (Max), maximum luminescence time (Time) and area under the curve of luminescence (AUC) (Kessi-Pérez et al., 2019a, b).

### Statistical Analysis

An analysis of variance (ANOVA) was used to compare the enological and kinetic parameters. The experimental mean values were statistically analyzed using Student’s *t*-test. The parameters associated with luminescence curves were compared using a non-parametric Kruskal–Wallis test. A *p*-value less than 0.05 (*p* < 0.05) was considered statistically significant.

## Results

### Deletion of the *GTR1* Gene Affects Nitrogen Consumption and Expression of Nitrogen Transporters Under Fermentation Conditions

**FIGURE 1:**
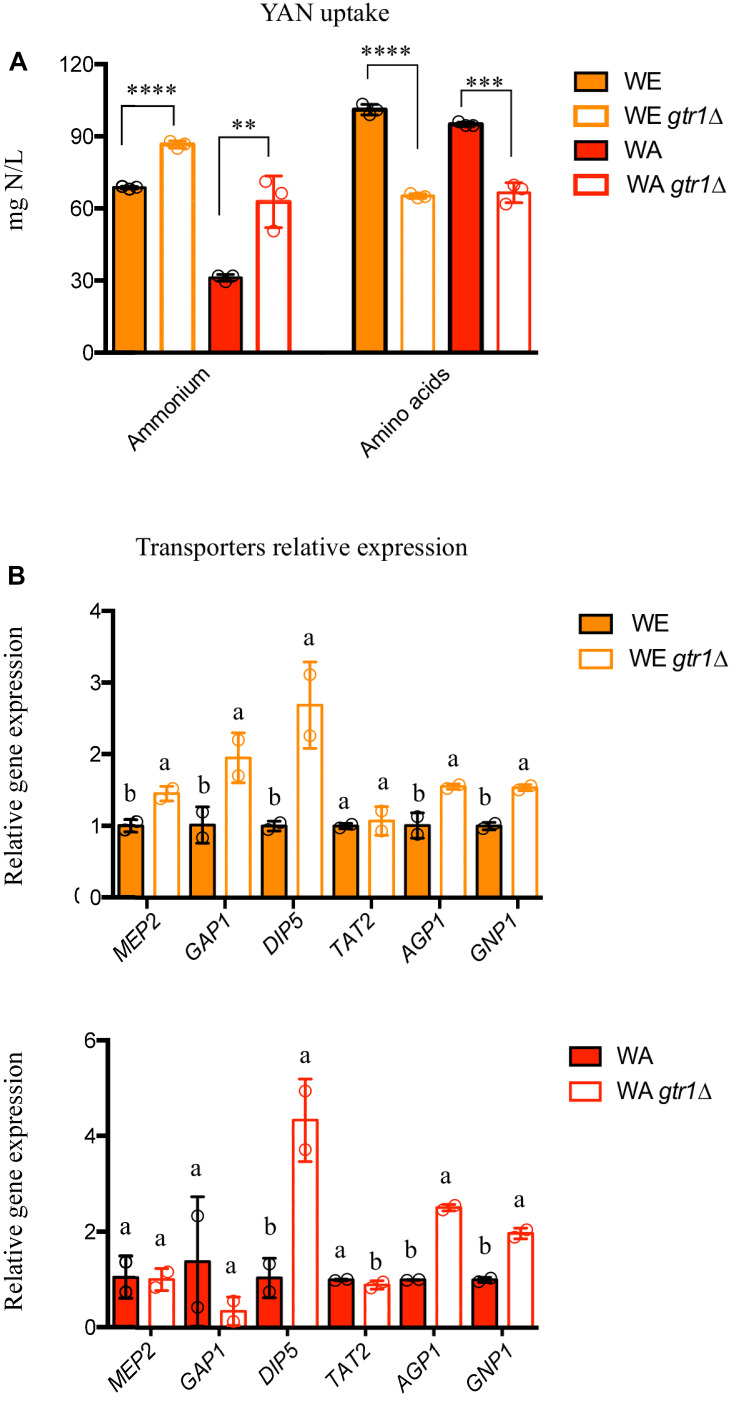
Fermentation performance of the *GTR1* null mutants in SM300 medium. **(A)** Nitrogen consumption for null mutants and wild type strains. Plotted values correspond to the average of three biological replicates (dots), with their standard error represented by bars (mean ± SE). The asterisks represent different levels of significance between the phenotypes of null mutants and wild type strains (*t*-test; ^∗∗^*p* < 0.01, ^∗∗∗^*p* < 0.001, and ^∗∗∗∗^*p* < 0.0001). **(B)** Relative gene expression at 6 h of fermentation for nitrogen transporters in the WE (top panel) and WA (bottom panel) genetic backgrounds. Expression levels were normalized using three housekeeping genes. Plotted values correspond to the average of two biological replicates (dots), with their standard error represented by bars (mean ± SE). Different letters represent significant statistical difference between the null mutants and wild type strains (Mood test with *p* < 0.05). WE: Wine/European; WA: West African.

Previously, we have demonstrated that a large allelic diversity exits within the TORC1 pathway and these allelic variants significantly impact nitrogen consumption. In this sense, *GTR1* allelic variants affect the consumption of amino acids and ammonium, where the Wine/European allele (WE) of this gene presented a preference for ammonium consumption, whereas the West African allele (WA) showed a preference for amino acids (Molinet et al., 2019). In this context, we assessed the role of the *GTR1* gene on nitrogen consumption under fermentation conditions, in a media with a high concentration of sugar and nitrogen as a limiting nutrient (SM300), in the WE and WA genetic backgrounds. Initially, we studied the phenotypic effects of *GTR1* deletion (*gtr1Δ)* in both strains. The deletion of the *GTR1* gene only increased the fermentative capacity by 22% in the WE genetic background, when compared to the wild type strain (Supplementary Figures S1, S2). However, both null mutants presented higher ammonium consumption, with a 21 and 50% difference between the null mutant strains and the respective wild type (WE and WA) strains (Figure 1A). In addition, *gtr1Δ* strains showed a lower overall amino acid consumption, with 35 and 30% differences between the null mutant strains and the respective wild type (WE and WA) strains (Figure 1A). The amino acids with statistically significant differences were aspartic acid, serine, glutamine, glycine, arginine, threonine, alanine, tyrosine, valine, isoleucine, leucine, and lysine (Supplementary Tables S3, S4). Despite this, null mutants improved the kinetic parameters resulting in a lower lag time and a higher efficiency than the wild type strains in micro-cultivation experiments in SM300 synthetic must (Supplementary Figure S3). In conclusion, the *GTR1* gene regulates nitrogen consumption under fermentation conditions affecting both the fermentative capacity and growth efficiency.

We then evaluated the relative gene expression of different nitrogen transporters (*MEP2*, *GAP1, DIP5, TAT2, AGP1*, and *GNP1*) regulated by TORC1 in the same set of strains (Figure 1B). Although, the WA strain presented higher expression levels of transporters than the WE strain (Supplementary Figure S4), this does not correlate with nitrogen consumption, since the WE strain takes up 55% more ammonium and 6% more amino acids than the WA strain (Figure 1A). Likewise, the deletion of *GTR1* in the WE genetic background presented higher expression levels in all the genes evaluated, except *TAT2* (Figure 1B). Similarly, the *gtr1Δ* strain showed higher gene expression of *DIP5, AGP1*, and *GNP1* in the WA genetic background (Figure 1B). Thus, the absence of the *GTR1* gene positively regulates the expression of nitrogen transporters.

### *GTR1* Deletion Decrease TORC1 Activation Under Fermentation Conditions

**FIGURE 2:**
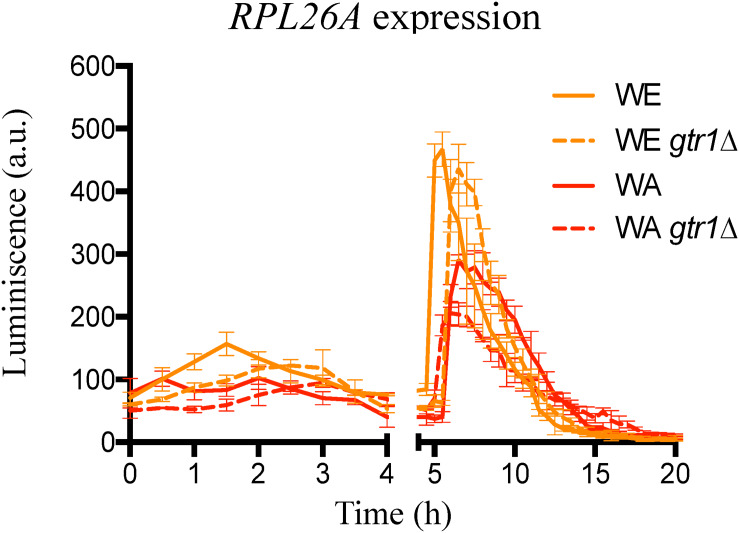
Indirect TORC1 activity in *GTR1* null mutants. Indirect monitoring of TORC1 activity was evaluated as luciferase expression controlled by *RPL26A* promoter under micro-culture conditions in SM300 medium until 20 h. Plotted values correspond to the average of three biological replicates, with their standard error represented by bars (mean ± SE). WE: Wine/European; WA: West African.

With the aim of evaluating the effect of *GTR1* deletion on TORC1 activity, we employed the expression of the *RPL26A* gene as an indirect readout for the activity status of TORC1. An active TORC1 leads to a transcriptional up-regulation of the *RPL26A* gene, whereas inactivation of TORC1 suppresses the transcription of *RPL26A* (Kessi-Pérez et al., 2019b). In this sense, we used the same strategy described by Kessi-Pérez et al. (2019b) and monitored the luminescence in strains containing the *Luc-URA3* reporter construct controlled by the *RPL26A* promoter in microculture conditions and in SM300 medium (Figure 2). In this condition, the behavior of the strains was similar to the one observed in nitrogen upshift experiments with two peaks of luciferase expression, one before 4 h and a second peak between 4 and 12 h (Kessi-Pérez et al., 2019b). However, in nitrogen upshift experiments, the WA strain presented a higher maximum luciferase expression than the WE strain, but in SM300 the WE strain had a greater maximum peak of expression (Figure 2), indicative of a possible higher TORC1 activation in the WE strain compared to the WA strain. Thus, the TORC1 activity under fermentation conditions (SM300) is different to that observed in laboratory media with a single nitrogen source. We should keep in mind that SM300 was made up of a complex mixture of ammonium and 19 amino acids that yeasts consume in sequential order of preference (Crépin et al., 2012).

Deletion of *GTR1* did not abolish the luminescence signal, which imply EGOC independent activation of the TORC1 pathway (Stracka et al., 2014). However, the results showed an effect in the TORC1 activity, where a lower peak of luminescence and a delay in the maximum luminescence were observed in the deletion strains compared to their wild type versions (Figure 2 and red values in Supplementary Table S2). In consequence, the differences observed in nitrogen consumption and expression of transporters between the null mutants (*gtr1Δ*) and their wild type strains could be the result of differences in the times and intensity of TORC1 activation. Altogether, these results confirmed the role of the *GTR1* gene on TORC1 activation in *S. cerevisiae* under fermentation conditions.

### SNPs Present in the *GTR1* Gene Affect Its Expression Levels During Alcoholic Fermentation

**FIGURE 3:**
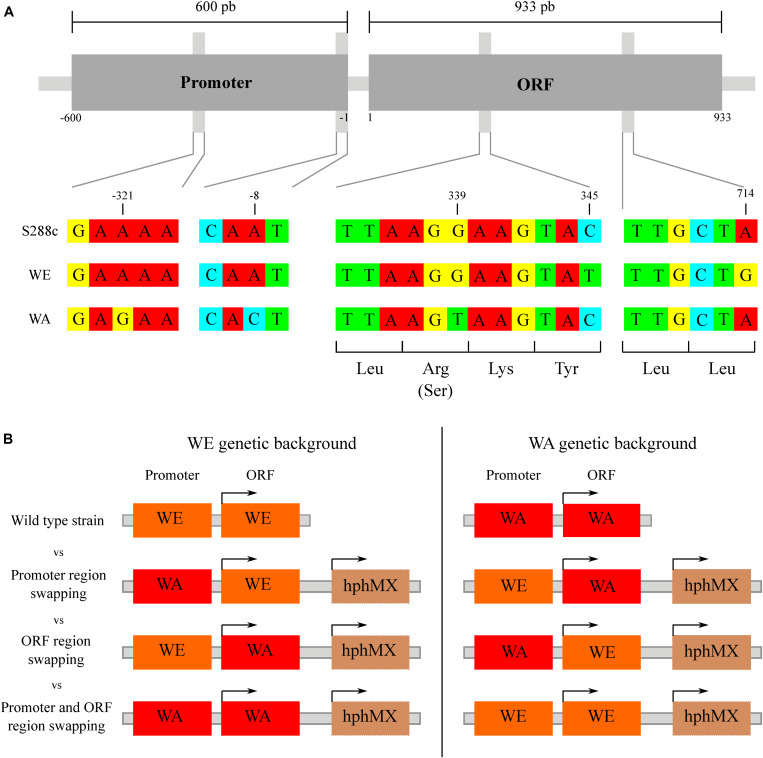
Nucleotide sequence variation for *GTR1* gene. **(A)** Alignment of the promoter and coding regions of the *GTR1* gene. The nucleotide sequence of S288c (reference), WE and WA strains were downloaded from the SGRP BLAST server of the University of Toronto (http://www.moseslab.csb.utoronto.ca/sgrp/) and aligned using MUSCLE (http://www.ebi.ac.uk/Tools/msa/muscle/). **(B)** Scheme of the swap strategy for promoter region, ORF region or both regions. Each promoter and/or ORF was swapped in the opposite genetic background, generating three different combinations. The strain background is shown as an orange (WE) or red (WA) rectangle.

**FIGURE 4:**
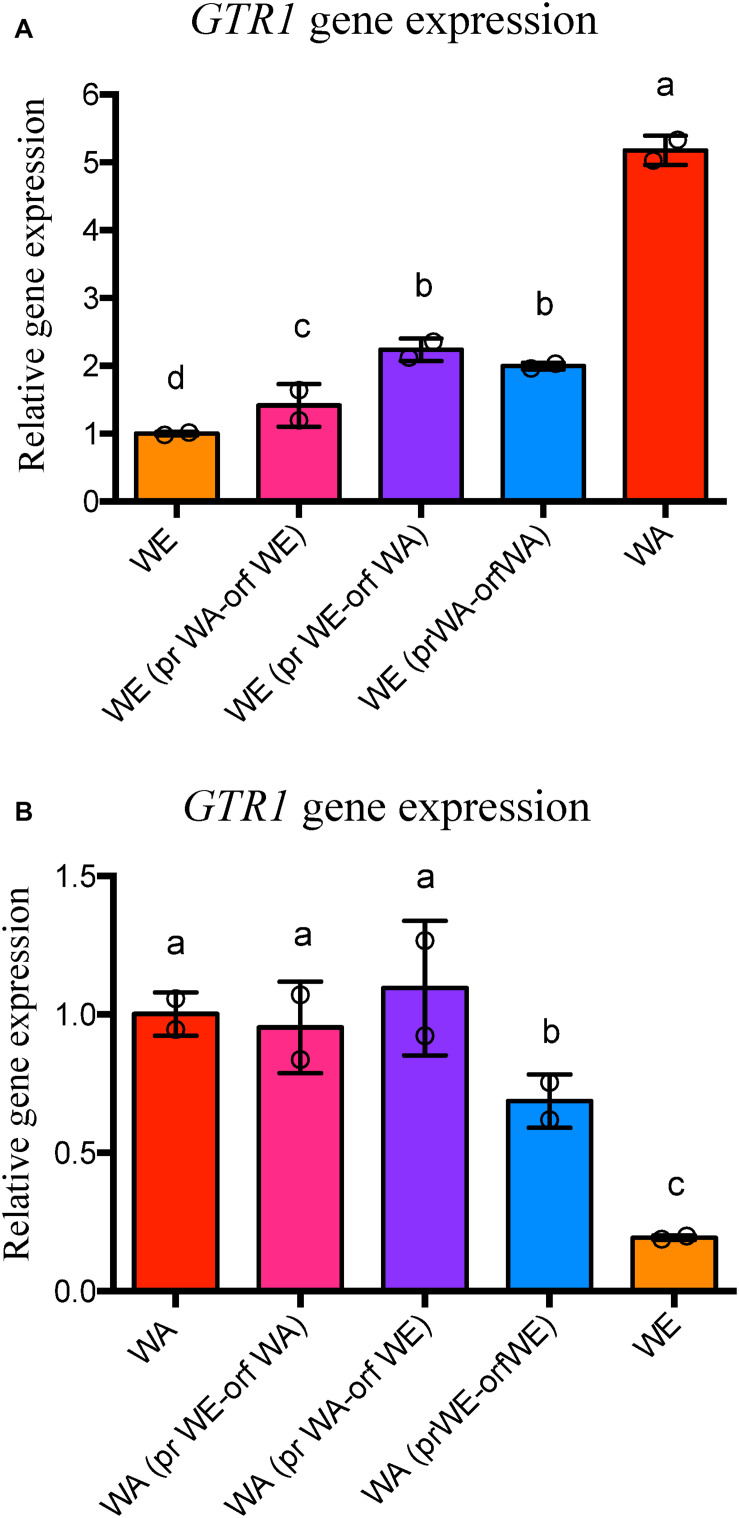
*GTR1* gene expression in strains with swapped regions. **(A)** Relative gene expression at 6 h of fermentation for the *GTR1* gene in the WE strains with swapped regions of *GTR1* coming from the WA strains. Expression levels are relative to the WE strain. **(B)** Relative gene expression at 6 h of fermentation for the *GTR1* gene in the WA strains with swapped regions of *GTR1* coming from the WE strains. Expression levels are relative to the WA strain. In both panels, expression levels were normalized using three housekeeping genes. Plotted values correspond to the average of two biological replicates (dots), with their standard error represented by bars (mean ± SE). Different letters represent significant statistical differences between the strains constructed and wild type strains (Mood test with *p* < 0.05). WE: Wine/European; WA, West African; pr, Promoter; orf, Open Reading Frame (ORF).

Previously, we determined that allelic diversity in the *GTR1* gene underlies nitrogen consumption differences between WE and WA strains (Molinet et al., 2019). This gene has three SNPs in the ORF of the WE and WA strains, two synonymous (C345T and A714G) in the WE strain and one non-synonymous (R113S) in the WA strain (Figure 3A). According to the SIFT prediction, although the non-synonymous mutation would be tolerated for protein function (Molinet et al., 2019), it may nevertheless cause an impact since this SNP is localized in the GTPase domain of the protein. With regards to the regulatory region, there are two SNPs in the region encompassing 600 bp upstream of the ATG codon: A-8C and A-321G present in the WA strain (Figure 3A). Considering that phenotypic differences between strains may be explained by polymorphisms in coding and/or non-coding regions (Thompson and Cubillos, 2017), we decided to determine the causal polymorphisms responsible for these phenotypic differences by an allele swapping strategy in the parental strains. We swapped the ORF, the regulatory region or both regions in the parental backgrounds (Figure 3B). We first determined the *GTR1* expression in all the modified strains at 6 h of fermentation (Figure 4). The WA strain showed a higher *GTR1* expression level compared to the WE strain due to a combination of cis and trans factors (Figure 4). These results point out that *GTR1* expression levels do not correlate with the TORC1 activation observed in the wild type strains (Figure 2), where WA strain showed lower TORC1 activation, suggesting an EGOC independent activation of TORC1 in the WE strain.

In the WE genetic background, we observed a higher *GTR1* expression level in the modified strains than in the WE wild type strain, with a greater effect for the polymorphisms inside the ORF region than the regulatory region of WA origin (Figure 4A). However, *GTR1* expression levels in the modified strains did not reach the level of the WA strain, highlighting the importance of trans factors and genetic interactions. On the other hand, in the WA genetic background, we observed differences in the expression levels only in the strain with both regions of WE origin, lower than the WA strain but higher than the WE strain (Figure 4B). These results indicate that not only the polymorphisms in the regulatory regions are important for *GTR1* gene expression, but also the polymorphisms in the coding region. As a result, SNPs present in the *GTR1* ORF of the WA strain affect *GTR1* expression in the WE genetic background, whereas the synergistic contribution of *GTR1* SNPs present in the ORF and regulatory region of the WE strain have a mild effect on *GTR1* expression in the WA genetic background.

### *GTR1* Coding Region From the WA Strain Modify TORC1 Activation in the WE Genetic Background

**FIGURE 5:**
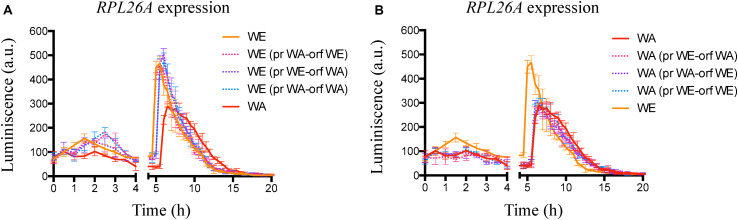
Indirect TORC1 activity in strains with *GTR1* swapped regions. **(A)** Indirect monitoring of TORC1 activity in the WE strains with swapped regions of *GTR1* coming from the WA strain, using micro culture conditions until 20 h. **(B)** Indirect monitoring of TORC1 activity in the WA strains with swapped regions of *GTR1* coming from the WE strain, using micro culture conditions until 20 h. Plotted values correspond to the average of three biological replicates, with their standard error represented by bars (mean ± SE). WE, Wine/European; WA, West African; pr, Promoter; orf: Open Reading Frame (ORF).

In order to evaluate the effect of polymorphisms present in the *GTR1* gene on TORC1 activity, we evaluated the TORC1 activation in the modified strains using the same indirect method previously assayed. In general, we observed a time delay in the maximum peak of luminescence in the modified WE strains which was similar to the WA strain, however, the intensity of the maximum peaks of luminescence were similar to the WE wild type strain (Figure 5A, blue values in Supplementary Table S2). During the first 4 h of growth, the WE strains with the promoter or both (promoter and ORF) regions of WA origin presented a delay in the maximum expression peak time with respect to the WE wild type strain (Figure 5A). In addition, the WE strain with the ORF of WA origin presented an intermediate phenotype between the WE and WA strains (Figure 5A), showing a delay in the time of the second peak, which correlates with the *GTR1* expression levels observed for this strain. Sequence analysis of 55 different strains of *S. cerevisiae* showed that the *GTR1* non-synonymous SNP is present only in strains from WA origin (Supplementary Figure S5), suggesting that this variant could be subject to selection in the palm wine ecological niche. Thus, this polymorphism has a greater probability of affecting the phenotype in strains of other ecological origins or phylogenetic clades. Finally, we did not observe differences in the TORC1 activity of the WA strain with the promoter region, coding sequence or both swapped regions of the *GTR1* gene coming from the WE strain (Figure 5B), indicative of a strong trans-regulation and genetic interaction in the WA strain.

### *GTR1* Coding Region From the WA Strain Impairs the Fermentative Performance in the WE Genetic Background

**FIGURE 6:**
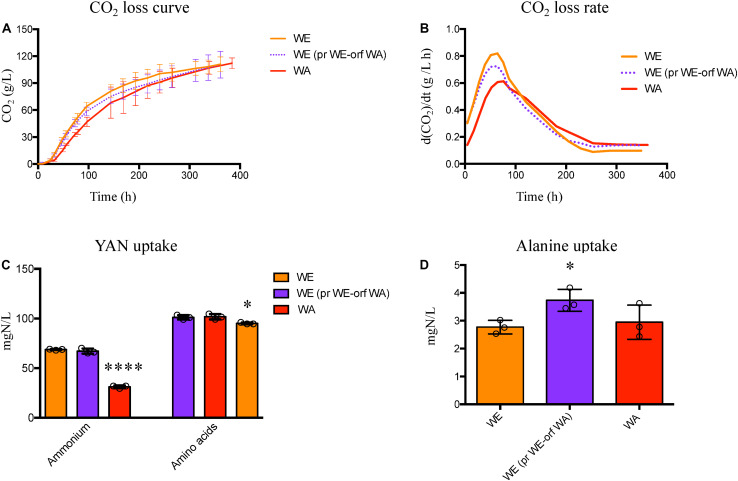
Fermentation performance for the WE strain with *GTR1* allele swapping. **(A)** Fermentative performance was evaluated by CO_2_ loss over the time and the kinetic parameters were extracted from the curves. **(B)** Maximum CO_2_ loss rate was determined by calculating the first derivate of the CO_2_ loss curve. **(C)** Ammonium and amino acids consumption for the WE strain with *GTR1* ORF swapping and the wild type parental strains. **(D)** Alanine consumption for the WE strain with *GTR1* ORF swapping and the wild type parental strains. Plotted values correspond to the average of three biological replicates (dots), with their standard error represented by bars (mean ± SE). The asterisks represent different levels of significance between the phenotypes of strains constructed and wild type strains (*t*-test; *p < 0.05 and ****p < 0.0001). WE, Wine/European; WA, West African; pr, Promoter; orf: Open Reading Frame (ORF).

**FIGURE 7:**
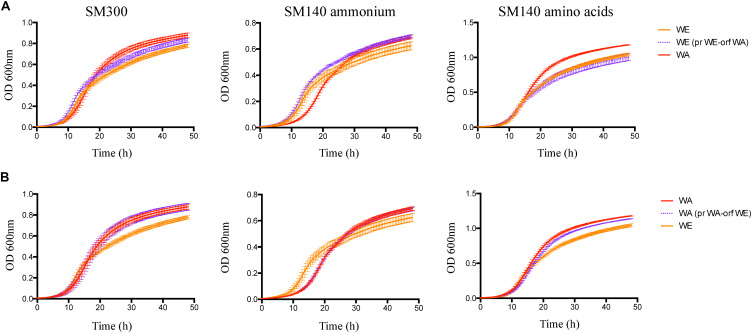
Growth performance for the WE and WA strains with *GTR1* allele swapping. **(A)** Growth curves for the strains with *GTR1* ORF swapping in WE genetic background, and WA genetic background **(B)**. Plotted values correspond to the average of three biological replicates, with their standard error represented by bars (mean ± SE). WE, Wine/European; WA, West African; pr, Promoter; orf, Open Reading Frame (ORF).

We then determined the effect of the *GTR1* SNPs on the fermentative kinetics and nitrogen consumption (Figure 6). In the WE genetic background, the phenotype of the strain with the WA *GTR1* ORF was intermediate between the two wild type strains (Figures 6A,B). We observed the same intermediary behavior in the growth kinetics in SM300 and SM140 with only ammonium or amino acids as a nitrogen source (Figure 7A and Table 1). In SM300 medium, we observed differences in the maximum specific growth rate and efficiency (Table 1), with intermediary values for the WE strain with the WA *GTR1* ORF, which correlates with the fermentative phenotype. These results highlight the importance of the SNPs in the coding region of *GTR1* for its biological function. Furthermore, considering our previous results in reciprocal hemizygote assays, the hemizygous strain with the WA allele had a preference for amino acid consumption, while the hemizygous strain with WE allele had a preference for ammonium consumption (Molinet et al., 2019), we decided to evaluate the growth kinetics in media containing only ammonium or amino acids as nitrogen sources (Figure 7 and Table 1). Results showed that the WE strain with a preference for ammonium consumption showed a higher maximum specific growth rate and a lower lag time in comparison to the WA strain in SM140-ammonium medium (Table 1). However, the WA strain showed higher growth efficiency in all the assayed culture conditions (Figure 7A). Interestingly, the WE strain with the WA ORF presented the same efficiency as the WA strain in SM140-ammonium (Figure 7A). On the contrary, in SM140-amino acids medium, the WE strain with the WA ORF presented the same efficiency as the WE strain (Figure 7A), but an intermediary value of maximum specific growth rate between WE and WA strains (Table 1). In addition, the previously described preference for amino acids consumption in the hemizygous strain with WA allele (Molinet et al., 2019) was confirmed by the higher efficiency and lower lag time for the WA strain in comparison to the WE strain in SM140-amino acids medium (Table 1). In summary, the strain with the WA ORF of the *GTR1* gene in the WE genetic background presented intermediary phenotypes between the wild type strains, confirming the effect of the coding region polymorphisms in the phenotypes evaluated.

**TABLE 1:** Kinetic parameters for strains with *GTR1* allele swapping in SM300, SM140-ammonium and SM140-amino acids culture conditions.

Strain	SM300			SM140-ammonium			SM140-amino acids		
	Lag (h)	μ_max_ (h^–1^)	Efficiency (ΔOD)	Lag (h)	μ_max_ (h^–1^)	Efficiency (ΔOD)	Lag (h)	μ_max_ (h^–1^)	Efficiency (ΔOD)
WE	2.26 ± 0.388	0.37 ± 10.008	0.772 ± 0.016	1.32 ± 0.183	0.319 ± 0.011	0.619 ± 0.029	2.74 ± 0.141	0.421 ± 0.374	1.040 ± 0.021
WE (prWA)	2.13 ± 0.496	0.380 ± 0.008	0.758 ± 0.054	1.35 ± 0.169	0.324 ± 0.006	0.651 ± 0.025	3.76 ± 0.679	0.374 ± 1.040	0.977 ± 0.013*
WE (orfWA)	2.03 ± 0.120	0.351 ± 0.009*	0.823 ± 0.020*	1.43 ± 0.327	0.310 ± 0.008	0.690 ± 0.012*	2.53 ± 0.070	0.387 ± 0.010*	0.997 ± 0.049
WE (pr-orfWA)	2.04 ± 0.276	0.362 ± 0.017	0.758 ± 0.073	1.79 ± 0.252	0.308 ± 0.012	0.669 ± 0.025	3.00 ± 1.031	0.409 ± 0.008	1.077 ± 0.046
WA	1.85 ± 0.098	0.323 ± 0.005	0.871 ± 0.020	2.52 ± 0.154	0.260 ± 0.002	0.687 ± 0.014	2.36 ± 0.133	0.351 ± 0.006	1.172 ± 0.005
WA (prWE)	1.69 ± 0.429	0.341 ± 0.009*	0.927 ± 0.021*	2.30 ± 0.229	0.254 ± 0.007	0.670 ± 0.052	2.60 ± 0.405	0.338 ± 0.007	1.149 ± 0.013*
WA (orf WE)	2.07 ± 0.294	0.325 ± 0.005	0.872 ± 0.031	2.06 ± 0.141	0.269 ± 0.004*	0.684 ± 0.014*	2.65 ± 0.188	0.328 ± 0.009*	1.131 ± 0.005***
WA (pr-orfWE)	1.95 ± 0.148	0.313 ± 0.003*	0.907 ± 0.022	2.48 ± 0.142	0.245 ± 0.001***	0.685 ± 0.025	2.82 ± 0.121*	0.330 ± 0.011*	1.170 ± 0.010

Values correspond to mean and SE of three biological replicates. Asterisks indicate significant differences between phenotypes of strains constructed and wild type strains (t-test, * p < 0.05 and *** p < 0.001). WE, Wine/European; WA, West African; orf, Open Reading Frame; pr: promoter.

We did not observe statistically significant differences in ammonium and amino acids consumption between the wild type and WE strain with the WA *GTR1* ORF (Figure 6C), but these strains showed an intermediate phenotype for alanine consumption (Figure 6D). This phenotypic difference is important considering the preference for ammonium consumption observed in the WE strain. Furthermore, when both regions of WA origin were present, we observed a higher consumption of alanine, valine, isoleucine, leucine and lysine in comparison to the WE wild type strain (Supplementary Table S3). Therefore, the effect of the *GTR1* polymorphisms on nitrogen consumption depends on genetic interactions, which is characteristic of complex traits (Nieduszynski and Liti, 2011; Swinnen et al., 2012).

Finally, in the WA genetic background, we observed a 21% difference in ammonium consumption between the strain with the WE *GTR1* ORF and its wild type strain (Supplementary Table S4). The modified strain presented a higher ammonium consumption, which correlates with the consumption of the WE wild type strain. We also evaluated the growth kinetics in SM300, SM140ammonium, and SM140-amino acids media (Figure 7B and Table 1), observing an intermediate efficiency between the two wild type strains in SM140-amino acids medium (Figure 7B). Altogether, these results highlight the importance of the SNPs in the *GTR1* coding region on the fermentative phenotype of *S. cerevisiae* and on its preference for the nitrogen source.

## Discussion

Studies in *S. cerevisiae* under fermentation conditions have shown that differences in nitrogen requirements between strains originate from variations in nitrogen sensing and signaling pathways (Brice et al., 2014b; Cubillos et al., 2017; Molinet et al., 2019). Previously, we identified and confirmed that allelic variants within the TORC1 pathway significantly impact nitrogen metabolism, influencing nitrogen consumption differences between strains representative of four clean lineages described in *S. cerevisiae* (Wine/European WE, West African WA, North American NA, and Sake SA). In particular, allelic variants of the *GTR1* gene affect the consumption of ammonium and amino acids. The WE allele of this gene presented a preference for ammonium consumption, while the WA allele had a preference for amino acids consumption (Molinet et al., 2019). In consequence, considering that regulation of TORC1 activity could be relevant to nitrogen consumption, we decided to further study the role of the *GTR1* gene on this phenotype in two different genetic backgrounds, providing evidence of the participation of this gene in nitrogen consumption and identifying relevant SNPs that affect TORC1 activation under fermentation conditions.

Null mutants of the *GTR1* gene took up more ammonium and less amino acid than the wild type strains in both genetic backgrounds. Furthermore, expression levels of the nitrogen transporters were higher in these null mutants (Figure 1). We did not observe correlation between amino acids consumption and expression levels of their respective transporters, possibly because gene expression and nitrogen consumption were measured at one single point during alcoholic fermentation. Thus, we cannot discard differences in gene expression throughout the fermentation process, where a hierarchical amino acids consumption was previously described (Crépin et al., 2012; Gutiérrez et al., 2016). Therefore, it would be relevant to study the kinetics of nitrogen consumption together with nitrogen transporters expression through the time course of fermentation.

Gtr1p participates in the EGO (Broach, 2012) and Gse complexes (Gao and Kaiser, 2006). The Gse complex regulates the Gap1p (general amino acid permease) intracellular sorting from late endosome to plasmatic membrane and Gap1p recycling is blocked in GSE mutants (Gao and Kaiser, 2006; MacDonald and Piper, 2017). Thus, in this complex, the *GTR1* deletion could be affecting amino acid consumption due to a decrease of Gap1p in the plasmatic membrane.

The EGO complex regulates TORC1 activity in response to nitrogen availability (Zhang et al., 2018). The addition of any amino acid to nitrogen-depleted cells was found to result in rapid but transient EGO-dependent TORC1 activation. However, longer TORC1 activation was only observed upon addition of a nitrogen source sustaining optimal growth, for example glutamine or ammonium, and in an EGO complex independent manner (Stracka et al., 2014). Thus, deletion of *GTR1* could transiently inactivate TORC1, activating the expression of genes regulated by NCR, such as ammonium permeases (*MEP1*, *MEP2*, and *MEP3*), and increasing ammonium consumption. This ammonium consumption could then activate TORC1 independently of the EGO complex, favoring the expression of some amino acid permeases (*AGP1, GNP1*, and *DIP5*). Thus, the deletion of *GTR1* affects cellular growth, where higher ammonium consumption increases growth efficiency and decreases lag time.

In this regard, we indirectly evaluated TORC1 activation using the expression of the *RPL26A* gene as readout for TORC1 activation (Kessi-Pérez et al., 2019b). This method was previously validated in four different strains representative of clean lineages described in *S. cerevisiae* (WE and WA background inclusive) and compared with traditional methods based on immunoblot of Sch9p and Rps6 phosphorylation, exposing strains a proline-to-glutamine upshift, where the maximum luminescence and the area under the curve obtained from graphics of absolute luminescence versus time resemble the results obtained by immunoblot of Sch9p and Rps6p (Kessi-Pérez et al., 2019a, b). Interestingly, the wild type strains showed the same behavior in nitrogen upshift experiments and in synthetic must (SM300), which simulates fermentation conditions. However, in nitrogen upshift experiments, the WA strain presented a higher maximum luciferase expression compared to the WE strain, but in SM300 the WE strain had a greater maximum peak of expression (Figure 2), indicating a possible higher TORC1 activation in SM300 in the WE strain. This behavior coincides with other phenotypes controlled by TORC1 activation such as chronological life span, where under fermentation conditions the role of Sir2p, Hst2p, Gcn5p, and Atg7 was the opposite to standard laboratory aging conditions (Orozco et al., 2012; Picazo et al., 2015). These results highlight the importance of evaluating TORC1 activity under fermentation conditions since its activity is dependent on the environment.

The *GTR1* deletion modify TORC1 activity with a lower peak of luminescence and maximum luminescence times higher than the wild type strains (Figure 2 and Supplementary Table S2). Thus, the differences observed in nitrogen consumption and expression of transporters between the *GTR1* null mutants and their wild type strains could be the consequence of difference in time and intensity in TORC1 activation. It has been reported that the inability of the Sake strain Kyokai n°7 (K7) to sporulate under nitrogen starvation conditions is due to a reduced TORC1 activity (Nakazawa et al., 2016). Consequently, a diminished TORC1 activity may have an impact on multiple phenotypes. Similarly, our results indicate a possible relationship between a diminished TORC1 activity and higher expression levels of nitrogen transporters, favoring ammonium consumption, but decreasing amino acid assimilation.

Since we previously described allelic variants of the *GTR1* gene that explain nitrogen consumption differences (Molinet et al., 2019), we sought to identify the SNPs responsible for these differences through an allele swapping approach in the parental strains. We swapped the ORF, the regulatory region or both regions in the parental backgrounds (Figure 3B). Initially, we determined *GTR1* expression in all the modified strains at 6 h of fermentation (Figure 4). Surprisingly, the WA strain presented a higher expression level compared to the WE strain, despite a lower TORC1 activity in this strain (Figure 2). Therefore, these results suggest that *GTR1* levels in the cell could affect TORC1 activity. It is important to mention that we did not evaluate the effect of hphMX on the phenotypes studied, thus we cannot discard cis effect and genetic interactions on the *GTR1* expression. However, the constructs were designed using the same orientation for the promoter, ORF and hphMX cassette, avoiding potential problems of convergent transcription and RNA polymerases collision (Pannunzio and Lieber, 2016).

In the case of the WE genetic background, we observed a higher impact in the studied phenotypes in the strain with the WA *GTR1* ORF. This strain presented higher expression levels of the *GTR1* gene compared to the wild type strain (Figure 4). In addition, it presented an intermediate TORC1 activity between the WE and WA strains (Figure 5). The WE strain with both regions (promoter and ORF) from WA origin presented a similar phenotype to the WE strain with only the ORF from WA origin during the second peak of luminescence, emphasizing the importance of the SNP present in the coding region of the GTR1 gene on the WE genetic background (Figure 5). Importantly, this non-synonymous SNP, R113S, is present only in strains from West African origin (Supplementary Figure S5), suggesting that this variant could be subject to selection in the palm wine ecological niche. However, we compared a very low number of sequences to validate this assumption and further analysis are necessary, including a higher number of strains used for palm wine fermentation. The R113S SNP is localized in the GTPase domain of the protein, where the GTP and GDP-bound conformations of Gtr1p are crucial for TORC1 activation and inactivation (Gong et al., 2011). Finally, the WE strain with the WA ORF of *GTR1* presented an intermediate fermentation and growth kinetic phenotypes with respect to the wild type strains in SM300 (Figures 6A,B, 7A). This confirms the effect of the polymorphisms in the *GTR1* coding region on the phenotypes evaluated.

In the case of the WA genetic background, we observed moderate effects on the studied phenotypes showing a greater *GTR1* expression compared to WE strain (Figure 4). Furthermore, the WA strain with the *GTR1* ORF and regulatory region coming from WE origin showed a slight but significant decrease in *GTR1* expression compared to the wild type WA strain (Figure 4). These results indicate the importance of the *GTR1* polymorphisms in the coding region on its own gene expression. Studies in different organisms have determined that the effect of synonymous SNPs in codon usage impact on nucleic acid stability, protein levels, protein structure and functions, even many human diseases have been associated to synonymous SNPs (Bali and Bebok, 2015).

Interestingly, we did not observe differences in the *RPL26A* expression profiles in the WA genetic background, indicative of strong trans-regulation and genetic interactions in the WA strain. Similar results were previously obtained studying the natural variation of the *GPD1* gene. Promoter swapping experiments between the evaluated strains showed different *GPD1* expression levels and times; however, no phenotypic differences were observed (glycerol yield) between wild type and modified strains (Tapia et al., 2018). Therefore, our results highlight how important the SNPs in the coding region of *GTR1* are to the fermentative phenotype of *S. cerevisiae*.

## Conclusion

In conclusion, we attempted to understand the role of the *GTR1* gene on nitrogen uptake during alcoholic fermentation in *S. cerevisiae*. In this regard, we observed in the *gtr1Δ* mutants a diminish TORC1 activity, an increase in expression levels of nitrogen transporters and ammonium consumption, but a decrease in the assimilation of amino acids. Furthermore, the non-synonymous polymorphism (R113S) present in the *GTR1* gene of the WA strain is relevant for TORC1 activity in the context of the WE strain. However, we were unable to identify mild phenotypic differences due to genetic interactions, suggesting further experiments are required to assess the phosphorylation levels of the proximal TORC1 targets or the GTPase activity of Gtr1p.

## Data Availability Statement

All datasets generated for this study are included in the article/Supplementary Material.

## Author Contributions

JM performed lab experiments and analyzed the experimental data. JM and FS prepared the manuscript. All authors designed and funded the research, discussed the results, read, and approved the final manuscript.

## Conflict of Interest

The authors declare that the research was conducted in the absence of any commercial or financial relationships that could be construed as a potential conflict of interest.

## Footnotes

1 http://www.moseslab.csb.utoronto.ca/sgrp/

2 http://www.ebi.ac.uk/Tools/msa/muscle/

## References

[B1] Bali V. Bebok Z. (2015). Decoding mechanisms by which silent codon changes influence protein biogenesis and function. *Int. J. Biochem. Cell Biol.* 64 58–74. 10.1016/j.biocel.2015.03.011 25817479 PMC4461553

[B2] Bell S.-J. Henschke P. A. (2005). Implications of nitrogen nutrition for grapes, fermentation and wine. *Aust. J. Grape Wine Res.* 11 242–295. 10.1111/j.1755-0238.2005.tb00028.x

[B3] Bely M. Sablayrolles J.-M. Barre P. (1990). Automatic detection of assimilable nitrogen deficiencies during alcoholic fermentation in oenological conditions. *J. Ferment. Bioeng.* 70 246–252. 10.1016/0922-338X(90)90057-4

[B4] Brice C. Cubillos F. A. Dequin S. Camarasa C. Martínez C. (2018). Adaptability of the *Saccharomyces cerevisiae* yeasts to wine fermentation conditions relies on their strong ability to consume nitrogen. *PLoS One* 13:e0192383. 10.1371/journal.pone.0192383 29432462 PMC5809068

[B5] Brice C. Sanchez I. Bigey F. Legras J. Blondin B. (2014a). A genetic approach of wine yeast fermentation capacity in nitrogen-starvation reveals the key role of nitrogen signaling. *BMC Genomics* 15:495. 10.1186/1471-2164-15-495 24947828 PMC4073503

[B6] Brice C. Sanchez I. Tesnière C. Blondin B. (2014b). Assessing the mechanisms responsible for differences between nitrogen requirements of *Saccharomyces cerevisiae* wine yeasts in alcoholic fermentation. *Appl. Environ. Microbiol.* 80 1330–1339. 10.1128/AEM.03856-13 24334661 PMC3911072

[B7] Broach J. R. (2012). Nutritional control of growth and development in yeast. *Genetics* 192 73–105. 10.1534/genetics.111.135731 22964838 PMC3430547

[B8] Conrad M. Schothorst J. Kankipati H. N. Van Zeebroeck G. Rubio-Texeira M. Thevelein J. M. (2014). Nutrient sensing and signaling in the yeast *Saccharomyces cerevisiae*. *FEMS Microbiol. Rev.* 38 254–299. 10.1111/1574-6976.12065 24483210 PMC4238866

[B9] Contreras A. García V. Salinas F. Urzúa U. Ganga M. A. Martínez C. (2012). Identification of genes related to nitrogen uptake in wine strains of *Saccharomyces cerevisiae*. *World J. Microbiol. Biotechnol.* 28 1107–1113. 10.1007/s11274-011-0911-3 22805832

[B10] Crépin L. Nidelet T. Sanchez I. Dequin S. Camarasa C. (2012). Sequential use of nitrogen compounds by *Saccharomyces cerevisiae* during wine fermentation: a model based on kinetic and regulation characteristics of Nitrogen permeases. *Appl. Environ. Microbiol.* 78 8102–8111. 10.1128/AEM.02294-12 22983966 PMC3485930

[B11] Crépin L. Sanchez I. Nidelet T. Dequin S. Camarasa C. (2014). Efficient ammonium uptake and mobilization of vacuolar arginine by *Saccharomyces cerevisiae* wine strains during wine fermentation. *Microb. Cell Fact.* 13:109. 10.1186/s12934-014-0109-0 25134990 PMC4244049

[B12] Crépin L. Truong N. M. Bloem A. Sanchez I. Dequin S. Camarasa C. (2017). Management of multiple nitrogen sources during wine fermentation by *Saccharomyces cerevisiae*. *Appl. Environ. Microbiol.* 83:AEM.02617-16. 10.1128/AEM.02617-16 28115380 PMC5311416

[B13] Cubillos F. Brice C. Molinet J. Tisné S. Abarca V. Tapia S. (2017). Identification of Nitrogen consumption genetic variants in yeast through QTL mapping and bulk segregant RNA-Seq analyses. *G3* 7 1693–1705. 10.1534/g3.117.042127 28592651 PMC5473750

[B14] Cubillos F. A. Louis E. J. Liti G. (2009). Generation of a large set of genetically tractable haploid and diploid *Saccharomyces* strains. *FEMS Yeast Res.* 9 1217–1225. 10.1111/j.1567-1364.2009.00583.x 19840116

[B15] De Virgilio C. Loewith R. (2006). Cell growth control: little eukaryotes make big contributions. *Oncogene* 25 6392–6415. 10.1038/sj.onc.1209884 17041625

[B16] Gao M. Kaiser C. A. (2006). A conserved GTPase-containing complex is required for intracellular sorting of the general amino-acid permease in yeast. *Nat. Cell Biol.* 8 657–667. 10.1038/ncb1419 16732272

[B17] Gómez-Alonso S. Hermosín-Gutiérrez I. García-Romero E. (2007). Simultaneous HPLC analysis of biogenic amines, amino acids, and ammonium ion as aminoenone derivatives in wine and beer samples. *J. Agric. Food Chem.* 55 608–613. 10.1021/jf062820m 17263449

[B18] Gong R. Li L. Liu Y. Wang P. Yang H. Wang L. (2011). Crystal structure of the Gtr1p-Gtr2p complex reveals new insights into the amino acid-induced TORC1 activation. *Genes Dev.* 25 1668–1673. 10.1101/gad.16968011 21816923 PMC3165931

[B19] Gutiérrez A. Chiva R. Sancho M. Beltran G. Arroyo-López F. N. Guillamon J. M. (2012). Nitrogen requirements of commercial wine yeast strains during fermentation of a synthetic grape must. *Food Microbiol.* 31 25–32. 10.1016/j.fm.2012.02.012 22475939

[B20] Gutiérrez A. Sancho M. Beltran G. Guillamon J. M. Warringer J. (2016). Replenishment and mobilization of intracellular nitrogen pools decouples wine yeast nitrogen uptake from growth. *Appl. Microbiol. Biotechnol.* 100 3255–3265. 10.1007/s00253-015-7273-y 26754818

[B21] Hatakeyama R. De Virgilio C. (2016). Unsolved mysteries of Rag GTPase signaling in yeast. *Small GTPases* 7 239–246. 10.1080/21541248.2016.1211070 27400376 PMC5129903

[B22] Ibstedt S. Stenberg S. Bagés S. Gjuvsland A. B. Salinas F. Kourtchenko O. (2015). Concerted Evolution of Life Stage Performances Signals Recent Selection on Yeast Nitrogen Use. *Mol. Biol. Evol.* 32 153–161. 10.1093/molbev/msu285 25349282

[B23] Jara M. Cubillos F. A. García V. Salinas F. Aguilera O. Liti G. (2014). Mapping genetic variants underlying differences in the central nitrogen metabolism in fermenter yeasts. *PLoS One* 9:e86533. 10.1371/journal.pone.0086533 24466135 PMC3897725

[B24] Kessi-Pérez E. I. Molinet J. Martínez C. (2020). Disentangling the genetic bases of *Saccharomyces cerevisiae* nitrogen consumption and adaptation to low nitrogen environments in wine fermentation. *Biol. Res.* 53 1–10. 10.1186/s40659-019-0270-3 31918759 PMC6950849

[B25] Kessi-Pérez E. I. Salinas F. González A. Su Y. Guillamón J. M. Hall M. N. (2019a). KAE1 allelic variants affect TORC1 activation and fermentation kinetics in *Saccharomyces cerevisiae*. *Front. Microbiol.* 10:1686. 10.3389/fmicb.2019.01686 31417508 PMC6685402

[B26] Kessi-Pérez E. I. Salinas F. Molinet J. González A. Muñiz S. Guillamón J. M. (2019b). Indirect monitoring of TORC1 signalling pathway reveals molecular diversity among different yeast strains. *Yeast* 36 65–74. 10.1002/yea.3351 30094872

[B27] MacDonald C. Piper R. C. (2017). Genetic dissection of early endosomal recycling highlights a TORC1-independent role for Rag GTPases. *J. Cell Biol.* 216 3275–3290. 10.1083/jcb.201702177 28768685 PMC5626546

[B28] Magasanik B. Kaiser C. A. (2002). Nitrogen regulation in *Saccharomyces cerevisiae*. *Gene* 290 1–18. 10.1016/S0378-1119(02)00558-9 12062797

[B29] Martínez C. Contreras A. Aguilera O. Ganga A. García V. (2014). The ICY1 gene from *Saccharomyces cerevisiae* affects nitrogen consumption during alcoholic fermentation. *Electron. J. Biotechnol.* 17 150–155. 10.1016/j.ejbt.2014.04.006

[B30] Martinez C. García V. González D. Jara M. Aguilera M. Ganga M. A. (2013). Gene expression of specific enological traits in wine fermentation. *Electron. J. Biotechnol.* 16 4–11. 10.2225/vol16-issue4-fulltext-8 27085523

[B31] Marullo P. Bely M. Masneuf-Pomarède I. Pons M. Aigle M. Dubourdieu D. (2006). Breeding strategies for combining fermentative qualities and reducing off-flavor production in a wine yeast model. *FEMS Yeast Res.* 6 268–279. 10.1111/j.1567-1364.2006.00034.x 16487348

[B32] Molinet J. Cubillos F. A. Salinas F. Liti G. Martínez C. (2019). Genetic variants of TORC1 signaling pathway affect nitrogen consumption in *Saccharomyces cerevisiae* during alcoholic fermentation. *PLoS One* 14:e0220515. 10.1371/journal.pone.0220515 31348805 PMC6660096

[B33] Nakazawa N. Sato A. Hosaka M. (2016). TORC1 activity is partially reduced under nitrogen starvation conditions in sake yeast Kyokai no. 7. *Saccharomyces cerevisiae*. *J. Biosci. Bioeng.* 121 247–252. 10.1016/j.jbiosc.2015.07.002 26272416

[B34] Nieduszynski C. A. Liti G. (2011). From sequence to function: Insights from natural variation in budding yeasts. *Biochim. Biophys. Acta* 1810 959–966. 10.1016/j.bbagen.2011.02.004 21320572 PMC3271348

[B35] Nissen T. L. Schulze U. Nielsen J. Villadsen J. (1997). Flux distributions in anaerobic, glucose-limited continuous cultures of *Saccharomyces cerevisiae*. *Microbiology* 143 203–218. 10.1099/00221287-143-1-203 9025295

[B36] Oldenburg K. R. Vo K. T. Michaelis S. Paddon C. (1997). Recombination-mediated PCR-directed plasmid construction in vivo in yeast. *Nucleic Acids Res.* 25 451–452. 10.1093/nar/25.2.451 9016579 PMC146432

[B37] Orozco H. Matallana E. Aranda A. (2012). Wine yeast sirtuins and Gcn5p control aging and metabolism in a natural growth medium. *Mech. Ageing Dev.* 133 348–358. 10.1016/j.mad.2012.03.013 22738658

[B38] Pannunzio N. R. Lieber M. R. (2016). Dissecting the roles of divergent and convergent transcription in chromosome instability. *Cell Rep.* 14 1025–1031. 10.1016/j.celrep.2015.12.098 26804908 PMC6028021

[B39] Pfaffl M. W. (2001). A new mathematical model for relative quantification in real-time RT-PCR. *Nucleic Acids Res.* 29:e45 10.1093/nar/29.9.e45 PMC55695 11328886

[B40] Picazo C. Orozco H. Matallana E. Aranda A. (2015). Interplay among Gcn5, Sch9 and mitochondria during chronological aging of wine yeast is dependent on growth conditions. *PLoS One* 10:e0117267. 10.1371/journal.pone.0117267 25658705 PMC4319768

[B41] Pretorius I. S. (2000). Tailoring wine yeast for the new millennium: novel approaches to the ancient art of winemaking. *Yeast* 16 675–729. 10.1002/1097-0061(20000615)16:8<675::AID-YEA585<3.0.CO;2-B 10861899

[B42] Rossignol T. Dulau L. Julien A. Blondin B. (2003). Genome-wide monitoring of wine yeast gene expression during alcoholic fermentation. *Yeast* 20 1369–1385. 10.1002/yea.1046 14663829

[B43] Salinas F. de Boer C. G. Abarca V. García V. Cuevas M. Araos S. (2016). Natural variation in non-coding regions underlying phenotypic diversity in budding yeast. *Sci. Rep.* 6:21849. 10.1038/srep21849 26898953 PMC4761897

[B44] Stracka D. Jozefczuk S. Rudroff F. Sauer U. Hall M. N. (2014). Nitrogen Source Activates TOR (Target of Rapamycin) Complex 1 via Glutamine and Independently of Gtr/Rag Proteins. *J. Biol. Chem.* 289 25010–25020. 10.1074/jbc.M114.574335 25063813 PMC4155668

[B45] Swinnen S. Thevelein J. M. Nevoigt E. (2012). Genetic mapping of quantitative phenotypic traits in *Saccharomyces cerevisiae*. *FEMS Yeast Res.* 12 215–227. 10.1111/j.1567-1364.2011.00777.x 22150948

[B46] Tapia S. M. Cuevas M. Abarca V. Delgado V. Rojas V. García V. (2018). GPD1 and ADH3 natural variants underlie glycerol yield differences in wine fermentation. *Front. Microbiol.* 9:1460. 10.3389/fmicb.2018.01460 30018610 PMC6037841

[B47] Tesnière C. Brice C. Blondin B. (2015). Responses of *Saccharomyces cerevisiae* to nitrogen starvation in wine alcoholic fermentation. *Appl. Microbiol. Biotechnol.* 99 7025–7034. 10.1007/s00253-015-6810-z 26201494

[B48] Teste M.-A. Duquenne M. François J. M. Parrou J.-L. (2009). Validation of reference genes for quantitative expression analysis by real-time RT-PCR in Saccharomyces cerevisiae. *BMC Mol. Biol.* 10:99 10.1186/1471-2199-10-99 PMC2776018 19874630

[B49] Thompson D. A. Cubillos F. A. (2017). Natural gene expression variation studies in yeast. *Yeast* 34 3–17. 10.1002/yea.3210 27668700

[B50] Vandesompele J. De Preter K. Pattyn F. Poppe B. Van Roy N. De Paepe A. (2002). Accurate normalization of real-time quantitative RT-PCR data by geometric averaging of multiple internal control genes. *Genome Biol.* 3:0034. 10.1186/gb-2002-3-7-research0034 12184808 PMC126239

[B51] Walker M. E. Nguyen T. D. Liccioli T. Schmid F. Kalatzis N. Sundstrom J. F. (2014). Genome-wide identification of the Fermentome; genes required for successful and timely completion of wine-like fermentation by *Saccharomyces cerevisiae*. *BMC Genomics* 15:552. 10.1186/1471-2164-15-552 24993029 PMC4099481

[B52] Warringer J. Blomberg A. (2003). Automated screening in environmental arrays allows analysis of quantitative phenotypic profiles in *Saccharomyces cerevisiae*. *Yeast* 20 53–67. 10.1002/yea.931 12489126

[B53] Yuan J. Reed A. Chen F. Stewart C. N. (2006). Statistical analysis of real-time PCR data. *BMC Bioinformatics* 7:85. 10.1186/1471-2105-7-85 16504059 PMC1395339

[B54] Zhang W. Du G. Zhou J. Chen J. (2018). Regulation of sensing, transportation, and catabolism of nitrogen sources in *Saccharomyces cerevisiae*. *Microbiol. Mol. Biol. Rev.* 82:e0040-17. 10.1128/MMBR.00040-17 29436478 PMC5813884

[B55] Zwietering M. H. Jongenburger I. L. Rombouts F. M. Van Riet K. (1990). Modeling of the bacterial growth curve. *Appl. Environ. Microbiol.* 56 1875–1881. 16348228 10.1128/aem.56.6.1875-1881.1990 PMC184525

