# Supplementary material for: GTR1 Affects Nitrogen Consumption and TORC1 Activity in Saccharomyces cerevisiae Under Fermentation Conditions
Source: Front Genet. 2020 May 25;11:519. doi: 10.3389/fgene.2020.00519 (PMC7261904; doi:10.3389/fgene.2020.00519)
Supplement: Supplementary file 1 [file Table_1.DOCX]

**Supplementary Table S1. List of primers used in this study.**

| **Gene** | **Description** | **Name** | **Oligonucleotide sequence (5’-3’)** | **Reference** |
| --- | --- | --- | --- | --- |
| *GTR1* | Promoter amplification | PR-F | GAT TAA GTT GGG TAA CGC CAG GGT TTT CCC AGT CAC GAC GTT GTA AAA CGC CAC GTG CTG GAA ATA ACA C | This study |
|  |  | PR-R | CGA TTT ACC GGA GCC GGA CCG GCC CAT CAG AAG CAG TTT CTT CCT ATT ATT TGA CGA CAT | This study |
|  | ORF amplification | ORF-F | ATG TCG TCA AAT AAT AGG AAG AAA CTG CTT CTG ATG GGC CGG TCC GGC TCC GGT AAA TCG | This study |
|  |  | ORF-R | GTC GAC CTG CAG CGT ACG AAG CTT CAG CTG GGT AAG AGA ACA TTT TAC AC | This study |
|  | Construction amplification | CON-F | TGC GGC GAA CCA TAA TCA ATT GCC GTC GCT AAT TTC CGC CCC ACG TGC TGG AAA TAA CAC | This study |
|  |  | CON-R | GTG CCT TAG CAT ACC TGG GAT CCG AAA GGG CCA TTG CAA CCA TAC GTT CCG GTA AGA GAA TAG TGG ATC TGA TAT CAT CG | This study |
| Hygromycin | HpH cassette amplification | HYG-F | AGC CAC CAC CGG TGT AAA ATG TTC TCT TAC CCA GCT GAA GCT TCG TAC GC | This study |
|  |  | HYG-R | GCG GAT AAC AAT TTC ACA CAG GAA ACA GCT ATG ACC ATG ATT ACG CCA AGT AGT GGA TCT GAT ATC ATC G | This study |
| *ACT1* | Primer real-time PCR | ACT1-F | TTG GCC GGT AGA GAT TTG AC | (Contreras et al., 2012) |
|  |  | ACT1-R | CCC AAA ACA GAA GGT GGA A | (Contreras et al., 2012) |
| *UBC6* | Primer real-time PCR | UBC6-F | GAT ACT TGG AAT CCT GGC TGG TCT GTC TC | (Teste et al., 2009) |
|  |  | UBC6-R | AAA GGG TCT TCT GTT TCA TCA CCT GTA TTT GC | (Teste et al., 2009) |
| *RPN2* | Primer real-time PCR | RPN2-F | GCG GAT ACA GGC ACA TTG GAT ACC | (Teste et al., 2009) |
|  |  | RPN2-R | TGT TGC TAC CTT CTC TAC CTC CTT ACC | (Teste et al., 2009) |
|  |  | MEP1-R | GGC GCC AGC GAT AAT ACT TAA | (Beltran et al., 2004) |
| *MEP2* | Primer real-time PCR | MEP2-F | GGT ATC ATC GCT GGC CTA GTG | (Beltran et al., 2004) |
|  |  | MEP2-R | ACA ACG GCT GAC CAG ATT GG | (Beltran et al., 2004) |
|  |  | MEP3-R | TTG TGC CGT CCA TTC CAA T | (Beltran et al., 2004) |
| *GAP1* | Primer real-time PCR | GAP1-F | CTG TGG ATG CTG CTG CTT CA | (Beltran et al., 2004) |
|  |  | GAP1-R | CAA CAC TTG GCA AAC CCT TGA | (Beltran et al., 2004) |
| *DIP5* | Primer real-time PCR | DIP5-F | TGG TTG CCA TTC AAA ACT CA | (Molinet et al., 2019) |
|  |  | DIP5-R | CGA AAA CCA AGA CAC AAG CA | (Molinet et al., 2019) |
| *TAT2* | Primer real-time PCR | TAT2-F | CTG GCC ACG TGC ATT GTC T | (Chiva et al., 2009) |
|  |  | TAT2-R | GCC TTC ATC GCC AGT CTA AAT C | (Chiva et al., 2009) |
| *AGP1* | Primer real-time PCR | AGP1-F | CGC CAT ATG TCA TTG CTG TTG | (Gutiérrez et al., 2012) |
|  |  | AGP-R | CAT GGA CAG CAC GGA AAG TAG A | (Gutiérrez et al., 2012) |
| *GNP1* | Primer real-time PCR | GNP1-F | TCG TGT GGT TCC TCA TTT CAT AA | (Chiva et al., 2009) |
|  |  | GNP1-R | CCG TTA GCA ACG GAA AGA ACA | (Chiva et al., 2009) |
|  |  | BAP2-R | TTC GTC CTC TTG TCT CAT TAG | (Saerens et al., 2008) |

Beltran, G., Novo, M., Rozes, N., Mas, A., and Guillamon, J. (2004). Nitrogen catabolite repression in during wine fermentations. *FEMS Yeast Res.* 4, 625–632. doi:10.1016/j.femsyr.2003.12.004.

Chiva, R., Baiges, I., Mas, A., and Guillamon, J. M. (2009). The role of *GAP1* gene in the nitrogen metabolism of *Saccharomyces cerevisiae* during wine fermentation. *J. Appl. Microbiol.* 107, 235–244. doi:10.1111/j.1365-2672.2009.04201.x.

Contreras, A., García, V., Salinas, F., Urzúa, U., Ganga, M. A., and Martínez, C. (2012). Identification of genes related to nitrogen uptake in wine strains of *Saccharomyces cerevisiae*. *World J. Microbiol. Biotechnol.* 28, 1107–1113. doi:10.1007/s11274-011-0911-3.

Gutiérrez, A., Chiva, R., Sancho, M., Beltran, G., Arroyo-López, F. N., and Guillamon, J. M. (2012). Nitrogen requirements of commercial wine yeast strains during fermentation of a synthetic grape must. *Food Microbiol.* 31, 25–32. doi:10.1016/j.fm.2012.02.012.

Molinet, J., Cubillos, F. A., Salinas, F., Liti, G., and Martínez, C. (2019). Genetic variants of TORC1 signaling pathway affect nitrogen consumption in *Saccharomyces cerevisiae* during alcoholic fermentation. *PLoS One* 14, e0220515. doi:10.1371/journal.pone.0220515.

Saerens, S. M. G., Verbelen, P. J., Vanbeneden, N., Thevelein, J. M., and Delvaux, F. R. (2008). Monitoring the influence of high-gravity brewing and fermentation temperature on flavour formation by analysis of gene expression levels in brewing yeast. *Appl. Microbiol. Biotechnol.* 80, 1039–1051. doi:10.1007/s00253-008-1645-5.

Teste, M.-A., Duquenne, M., François, J. M., and Parrou, J.-L. (2009). Validation of reference genes for quantitative expression analysis by real-time RT-PCR in *Saccharomyces cerevisiae*. *BMC Mol. Biol.* 10, 99. doi:10.1186/1471-2199-10-99.
