# Supplementary material for: GTR1 Affects Nitrogen Consumption and TORC1 Activity in Saccharomyces cerevisiae Under Fermentation Conditions
Source: Front Genet. 2020 May 25;11:519. doi: 10.3389/fgene.2020.00519 (PMC7261904; doi:10.3389/fgene.2020.00519)
Supplement: Supplementary file 2 [file Table_2.DOCX]

**Supplementary Table S2. Indirect TORC1 activation extracted from luminescence curves.**

| **Strain** | **Max_0-12_ (a.u.)** | **Time_0-12_ (h)** | **AUC_0-12_ (a.u.)** | **Max_0-4_ (a.u.)** | **Time_0-4_ (h)** | **AUC_0-4_ (a.u.)** |
| --- | --- | --- | --- | --- | --- | --- |
| WE | 476 ± 14 | 5.3 ± 0.3 | 2,116 ± 175 | 157 ± 19 | 1.5 ± 0.0 | 442 ± 20 |
| WE *gtr1*Δ | 456 ± 5.6 | **6.7 ± 0.3*** | 2,040 ± 91 | 134 ± 15 | **2.3 ± 0.6*** | **375 ± 12*** |
| WE (prWA) | 466 ± 17 | 5.5 ± 0.0 | 2,176 ± 73 | **176 ± 4*** | **2.7 ± 0.3*** | **477 ± 9*** |
| WE (orfWA) | 506 ± 22 | **6.0 ± 0.0*** | 2,155 ± 132 | 152 ± 18 | 2.0 ± 0.5 | 437 ± 41 |
| WE (pr-orfWA) | **493 ± 3.2*** | 5.8 ± 0.3 | 2,222 ± 37 | 183 ± 18 | **2.5 ± 0.0*** | 478 ± 44 |
| WA | 298 ± 8 | 7.0 ± 0.9 | 1,730 ± 19 | 109 ± 12 | 0.8 ± 1.0 | 324 ± 22 |
| WA *gtr1*Δ | **217 ± 8*** | 6.5 ± 0.5 | **1,345 ± 52*** | 95 ± 7 | **3.0 ± 0.0*** | 281 ± 18 |
| WA (prWE) | 288 ± 12 | 6.8 ± 1.0 | 1,615 ± 121 | 103 ± 10 | 2.3 ± 1.2 | 304 ± 28 |
| WA (orf WE) | 296 ± 17 | 6.7 ± 0.6 | 1,577 ± 33* | 107 ± 10 | 1.3 ± 0.8 | 308 ± 30 |
| WA (pr-orfWE) | 310 ± 5 | 6.3 ± 0.3 | 1,713 ± 53 | 108 ± 6 | 1.5 ± 0.9 | 327 ± 14 |

Values correspond to mean and SE of three biological replicas. Max: maximum luminescence; Time: maximum luminescence time; AUC: area under the curve of luminescence; 0-12: 0-12 hours’ interval; 0-4: 0-4 hours’ interval. Asterisks indicate significant differences between phenotypes of parental strains (WE or WA) with their respective mutants (Kruskal-Wallis test, p<0.05). WE: Wine/European; WA: West African; orf: Open Reading Frame; pr: promoter. Red values represent statistically significant differences between *gtr1*Δ mutants and wild type strains, and blue values represent statistically significant differences between strains with different construct and wild type strains.
