# Supplementary material for: GTR1 Affects Nitrogen Consumption and TORC1 Activity in Saccharomyces cerevisiae Under Fermentation Conditions
Source: Front Genet. 2020 May 25;11:519. doi: 10.3389/fgene.2020.00519 (PMC7261904; doi:10.3389/fgene.2020.00519)
Supplement: Supplementary file 3 [file Table_3.DOCX]

**Supplementary Table S3. Nitrogen consumption (mgN/L) of mutants in the WE genetic background.**

| **Nitrogen source** | **WE** | **WE *gtr1*Δ** | **p-value** | **WE (pWA-oWE)** | **p-value** | **WE (pWE-oWA)** | **p-value** | **WE (pWA-oWA)** | **p-value** |
| --- | --- | --- | --- | --- | --- | --- | --- | --- | --- |
| Aspartic | 3.484 ± 0.062 | 2.294 ± 0.058 | **< 0.0001** | 3.558 ± 0.070 | 0.2434 | 3.491 ± 0.064 | 0.8979 | 3.530 ± 0.112 | 0.5715 |
| Glutamic | 4.874 ± 0.183 | 2.590 ± 0.029 | **< 0.0001** | 5.374 ± 0.320 | 0.0784 | 4.858 ± 0.224 | 0.9291 | 5.314 ± 0.356 | 0.1299 |
| Serine | 6.668 ± 0.129 | 2.598 ± 0.089 | **< 0.0001** | 6.867 ± 0.171 | 0.1822 | 6.721 ± 0.158 | 0.6745 | 6.730 ± 0.173 | 0.6446 |
| Histidine | 2.510 ± 0.015 | 2.006 ± 0.034 | **< 0.0001** | 2.626 ± 0.051 | **0.0191** | 2.539 ± 0.053 | 0.4153 | 2.566 ± 0.077 | 0.2870 |
| Glutamine | 29.306 ± 0.655 | 17.616 ± 0.461 | **< 0.0001** | 29.258 ± 0.386 | 0.9182 | 29.078 ± 0.491 | 0.6559 | 28.675 ± 0.063 | 0.1724 |
| Glycine | 0.205 ± 0.019 | -0.756 ± 0.021 | **< 0.0001** | 0.533 ± 0.127 | **0.0113** | 0.353 ± 0.064 | **0.0184** | 0.533 ± 0.065 | **0.0011** |
| Arginine | 5.090 ± 0.687 | 3.781 ± 0.422 | **0.0482** | 6.509 ± 0.913 | 0.0979 | 4.771 ± 0.997 | 0.6718 | 5.642 ± 0.379 | 0.2899 |
| Threonine | 6.677 ± 0.112 | 3.050 ± 0.072 | **< 0.0001** | 6.752 ± 0.090 | 0.4177 | 6.689 ± 0.109 | 0.9021 | 6.708 ± 0.097 | 0.7306 |
| Alanine | 2.771 ± 0.244 | -6.398 ± 0.286 | **< 0.0001** | 5.084 ± 1.001 | **0.0177** | 3.734 ± 0.395 | **0.0229** | 5.409 ± 0.904 | **0.0082** |
| Tyrosine | 0.975 ± 0.016 | 0.839 ± 0.020 | **0.0008** | 1.027 ± 0.038 | 0.0973 | 0.955 ± 0.020 | 0.2420 | 1.052 ± 0.052 | 0.0717 |
| Valine | 4.642 ± 0.059 | 3.946 ± 0.025 | **< 0.0001** | 4.834 ± 0.148 | 0.1054 | 4.614 ± 0.052 | 0.5725 | 4.942 ± 0.135 | **0.0243** |
| Methionine | 2.416 ± 0.000 | 2.416 ± 0.000 | 1.0000 | 2.416 ± 0.000 | 1.0000 | 2.416 ± 0.000 | 1.0000 | 2.416 ± 0.000 | 1.0000 |
| Cysteine | 0.255 ± 0.175 | 0.000 ± 0.141 | 0.1208 | 0.681 ± 0.290 | 0.0954 | 0.143 ± 0.277 | 0.5851 | 0.348 ± 0.099 | 0.4692 |
| Isoleucine | 3.563 ± 0.011 | 3.388 ± 0.006 | **< 0.0001** | 3.577 ± 0.006 | 0.1161 | 3.563 ± 0.011 | 1.0000 | 3.588 ± 0.006 | **0.0249** |
| Leucine | 7.051 ± 0.000 | 7.041 ± 0.000 | **< 0.0001** | 7.066 ± 0.006 | **0.0161** | 7.058 ± 0.012 | 0.3739 | 7.069 ± 0.006 | **0.0075** |
| Phenilalanine | 19.087 ± 0.017 | 19.073 ± 0.010 | 0.2794 | 19.107 ± 0.005 | 0.1242 | 19.101 ± 0.013 | 0.3153 | 19.109 ± 0.005 | 0.0907 |
| Lysine | 1.550 ± 0.051 | 1.745 ± 0.012 | **0.0030** | 1.722 ± 0.029 | **0.0072** | 1.679 ± 0.077 | 0.0741 | 1.745 ± 0.044 | **0.0074** |
| Ammonium | 68.666 ± 0.527 | 86.639 ± 1.471 | **< 0.0001** | 72.692 ± 2.350 | **0.0443** | 67.103 ± 2.561 | 0.3588 | 66.229 ± 5.017 | 0.4499 |
| Total amino acids | 101.123 ± 2.223 | 65.229 ± 0.952 | **< 0.0001** | 106.789 ± 3.440 | 0.0682 | 101.763 ± 2.678 | 0.7661 | 105.377 ± 1.860 | 0.06906 |

Student’s t-test was performed. Probability values lower than 0.05 were considered significantly different from WE parental strain.
