# Supplementary material for: GTR1 Affects Nitrogen Consumption and TORC1 Activity in Saccharomyces cerevisiae Under Fermentation Conditions
Source: Front Genet. 2020 May 25;11:519. doi: 10.3389/fgene.2020.00519 (PMC7261904; doi:10.3389/fgene.2020.00519)
Supplement: Supplementary file 4 [file Table_4.DOCX]

**Supplementary Table S4. Nitrogen consumption (mgN/L) of mutants in the WA genetic background.**

| **Nitrogen source** | **WA** | **WA *gtr1*Δ** | **p-value** | **WA (pWE-oWA)** | **p-value** | **WA (pWA-oWE)** | **p-value** | **WA (pWE-oWE)** | **p-value** |
| --- | --- | --- | --- | --- | --- | --- | --- | --- | --- |
| Aspartic | 2.777 ± 0.107 | 1.447 ± 0.104 | **0.001** | 2.704 ± 0.046 | 0.3340 | 2.728 ± 0.006 | 0.4710 | 2.630 ± 0.141 | 0.2236 |
| Glutamic | 2.334 ± 0.119 | 1.346 ± 0.736 | 0.0832 | 2.552 ± 0.210 | 0.1920 | 2.062 ± 0.302 | 0.2195 | 2.350 ± 0.152 | 0.8936 |
| Serine | 5.899 ± 0.240 | 1.948 ± 0.294 | **< 0.0001** | 5.730 ± 0.112 | 0.3318 | 5.912 ± 0.023 | 0.9284 | 5.477 ± 0.359 | 0.1666 |
| Histidine | 2.073 ± 0.072 | 1.776 ± 0.176 | 0.0540 | 2.944 ± 0.596 | 0.0658 | 2.613 ± 0.496 | 0.1355 | 2.142 ± 0.185 | 0.5777 |
| Glutamine | 28.416 ± 0.926 | 18.470 ± 0.533 | **< 0.0001** | 26.893 ± 1.076 | 0.1366 | 27.958 ± 0.872 | 0.5669 | 26.912 ± 1.037 | 0.1341 |
| Glycine | 0.645 ± 0.131 | 1.042 ± 0.056 | **0.0084** | 0.732 ± 0.084 | 0.3877 | 0.781 ± 0.226 | 0.4170 | 0.676 ± 0.011 | 0.7031 |
| Arginine | 4.016 ± 0.206 | 6.192 ± 0.490 | **0.0021** | 4.586 ± 0.691 | 0.2427 | 4.340 ± 1.061 | 0.6315 | 4.263 ± 0.149 | 0.1680 |
| Threonine | 6.736 ± 0.226 | 1.764 ± 0.264 | **< 0.0001** | 6.480 ± 0.178 | 0.1980 | 6.645 ± 0.126 | 0.5774 | 6.323 ± 0.283 | 0.1196 |
| Alanine | 2.946 ± 0.614 | 0.183 ± 1.164 | **0.0220** | 3.198 ± 0.572 | 0.6316 | 2.889 ± 1.095 | 0.9405 | 3.140 ± 0.366 | 0.6633 |
| Tyrosine | 0.728 ± 0.008 | 0.330 ± 0.049 | **0.0002** | 0.730 ± 0.019 | 0.8416 | 0.712 ± 0.054 | 0.6499 | 0.753 ± 0.049 | 0.4199 |
| Valine | 4.750 ± 0.059 | 1.902 ± 0.196 | **< 0.0001** | 4.758 ± 0.055 | 0.8722 | 4.670 ± 0.169 | 0.4813 | 4.810 ± 0.150 | 0.5545 |
| Methionine | 2.453 ± 0.000 | 2.353 ± 0.128 | 0.2463 | 2.453 ± 0.000 | 1.0000 | 2.453 ± 0.000 | 1.0000 | 2.453 ± 0.000 | 1.0000 |
| Cysteine | -0.039 ± 0.077 | 0.472 ± 0.347 | 0.0675 | 0.255 ± 0.192 | 0.0695 | -0.166 ± 0.241 | 0.4326 | 0.039 ± 0.116 | 0.3914 |
| Isoleucine | 3.581 ± 0.012 | 2.047 ± 0.124 | **< 0.0001** | 3.585 ± 0.011 | 0.7247 | 3.567 ± 0.043 | 0.6119 | 3.595 ± 0.028 | 0.4685 |
| Leucine | 7.242 ± 0.016 | 6.457 ± 0.074 | **< 0.0001** | 7.235 ± 0.016 | 0.6213 | 7.224 ± 0.038 | 0.4929 | 7.235 ± 0.012 | 0.5790 |
| Phenilalanine | 18.642 ± 0.013 | 16.965 ± 0.141 | **< 0.0001** | 18.577 ± 0.047 | 0.0829 | 18.622 ± 0.084 | 0.7063 | 18.582 ± 0.087 | 0.3055 |
| Lysine | 1.862 ± 0.012 | 1.898 ± 0.013 | **0.0249** | 1.854 ± 0.012 | 0.4676 | 1.836 ± 0.028 | 0.2144 | 1.847 ± 0.051 | 0.6388 |
| Ammonium | 31.152 ± 1.385 | 62.784 ± 10.747 | **0.0072** | 28.558 ± 1.219 | 0.0715 | 39.352 ± 1.552 | **0.0024** | 29.990 ± 6.050 | 0.7621 |
| Total amino acids | 95.061 ± 0.910 | 66.592 ± 4.182 | **0.0003** | 95.265 ± 1.120 | 0.8182 | 94.847 ± 2.777 | 0.9052 | 93.227 ± 1.726 | 0.1789 |

Student’s t-test was performed. Probability values lower than 0.05 were considered significantly different from WA parental strain.
