# Supplementary figures and images for: GTR1 Affects Nitrogen Consumption and TORC1 Activity in Saccharomyces cerevisiae Under Fermentation Conditions
Source: Front Genet. 2020 May 25;11:519. doi: 10.3389/fgene.2020.00519 (PMC7261904; doi:10.3389/fgene.2020.00519)

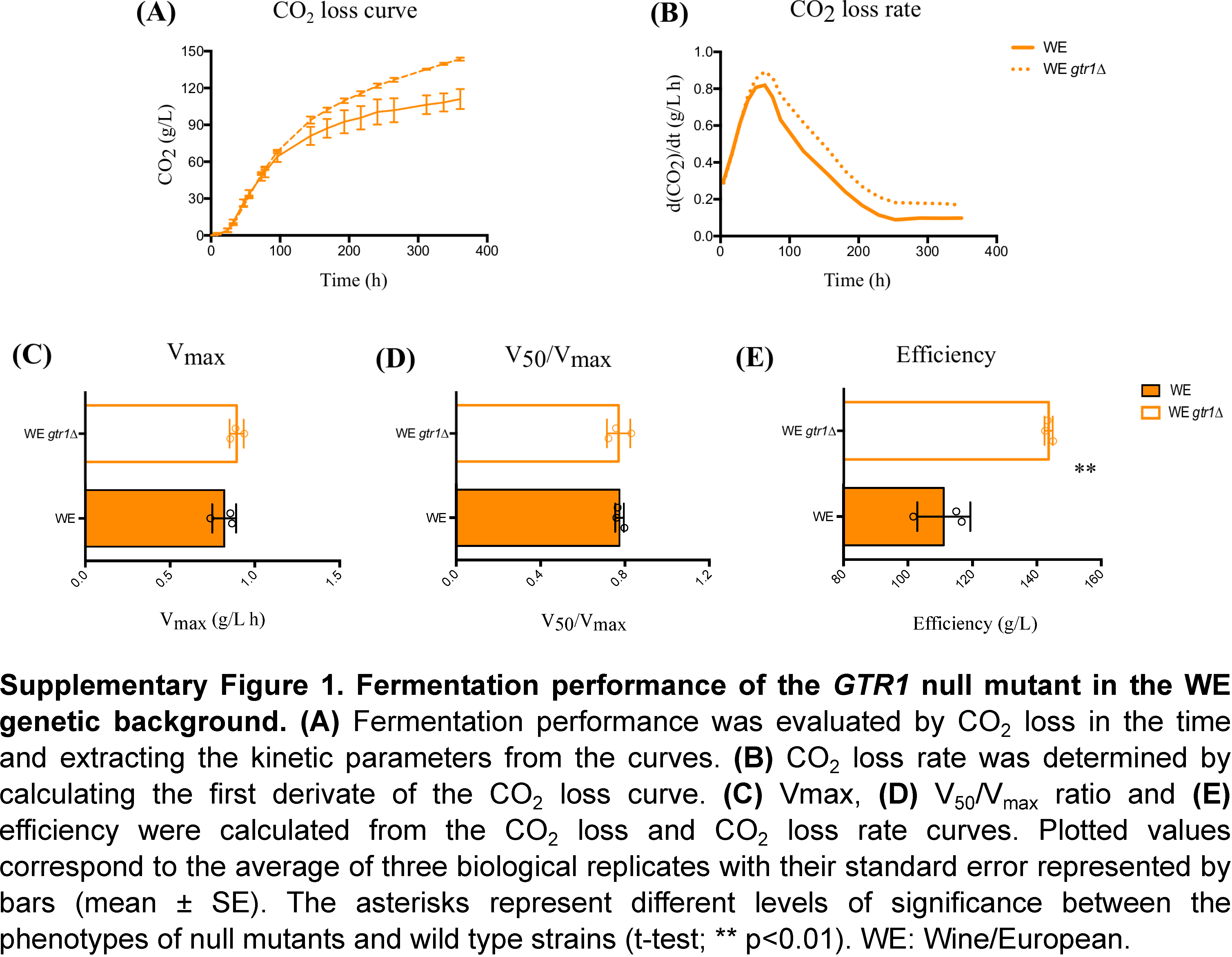

Supplement: Supplementary file 5 [file Image_1.TIF]

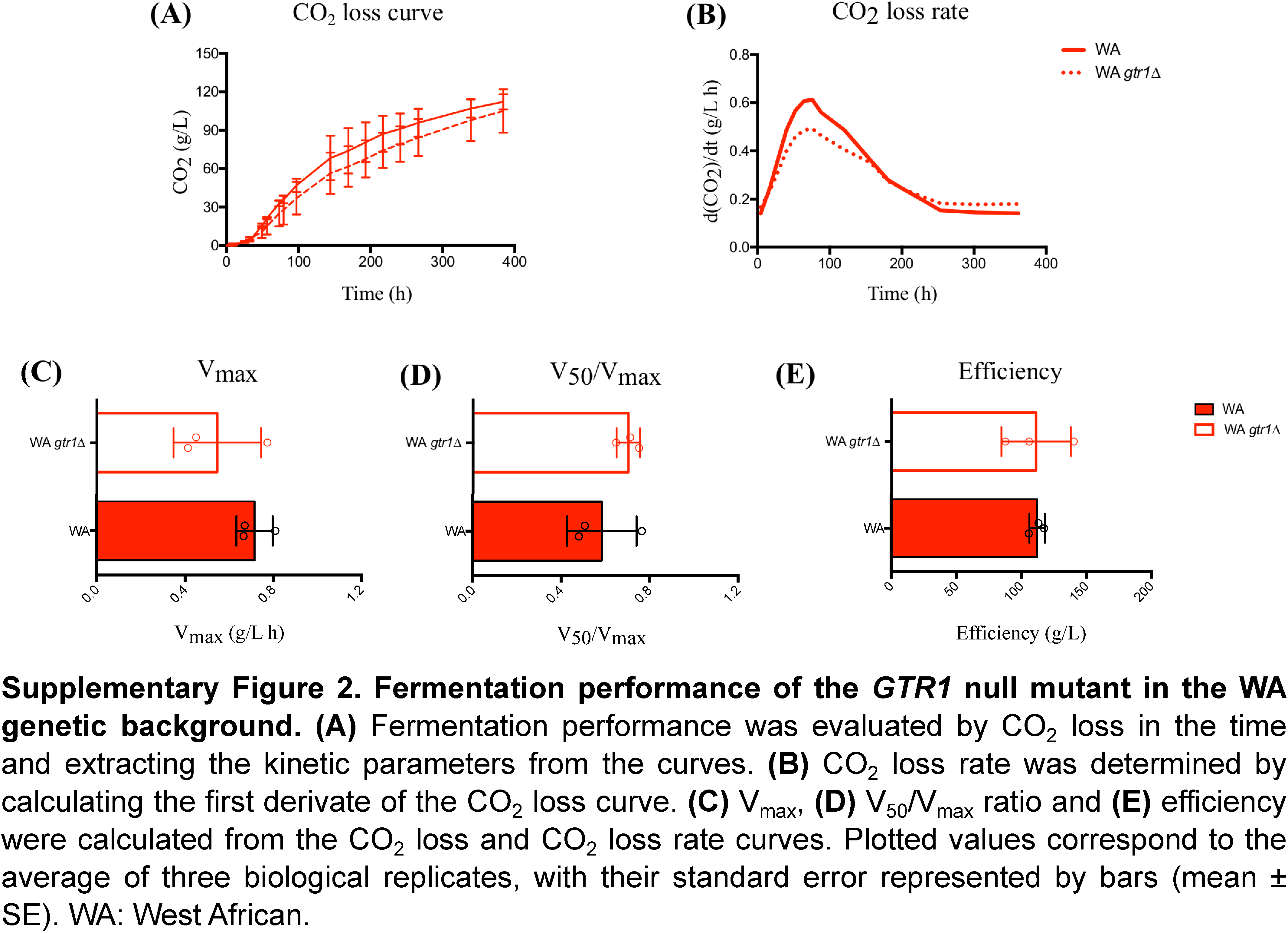

Supplement: Supplementary file 6 [file Image_2.TIF]

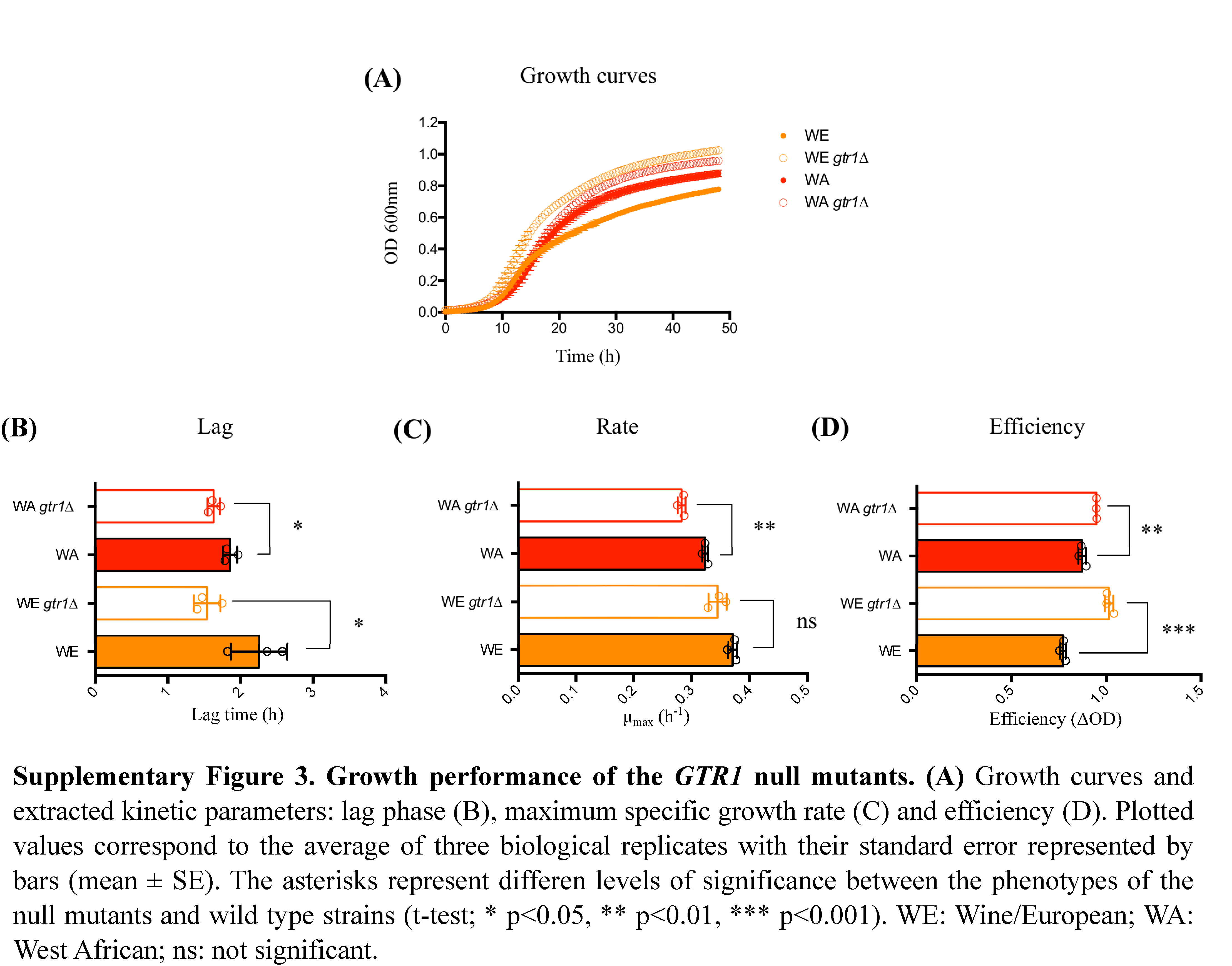

Supplement: Supplementary file 7 [file Image_3.TIF]

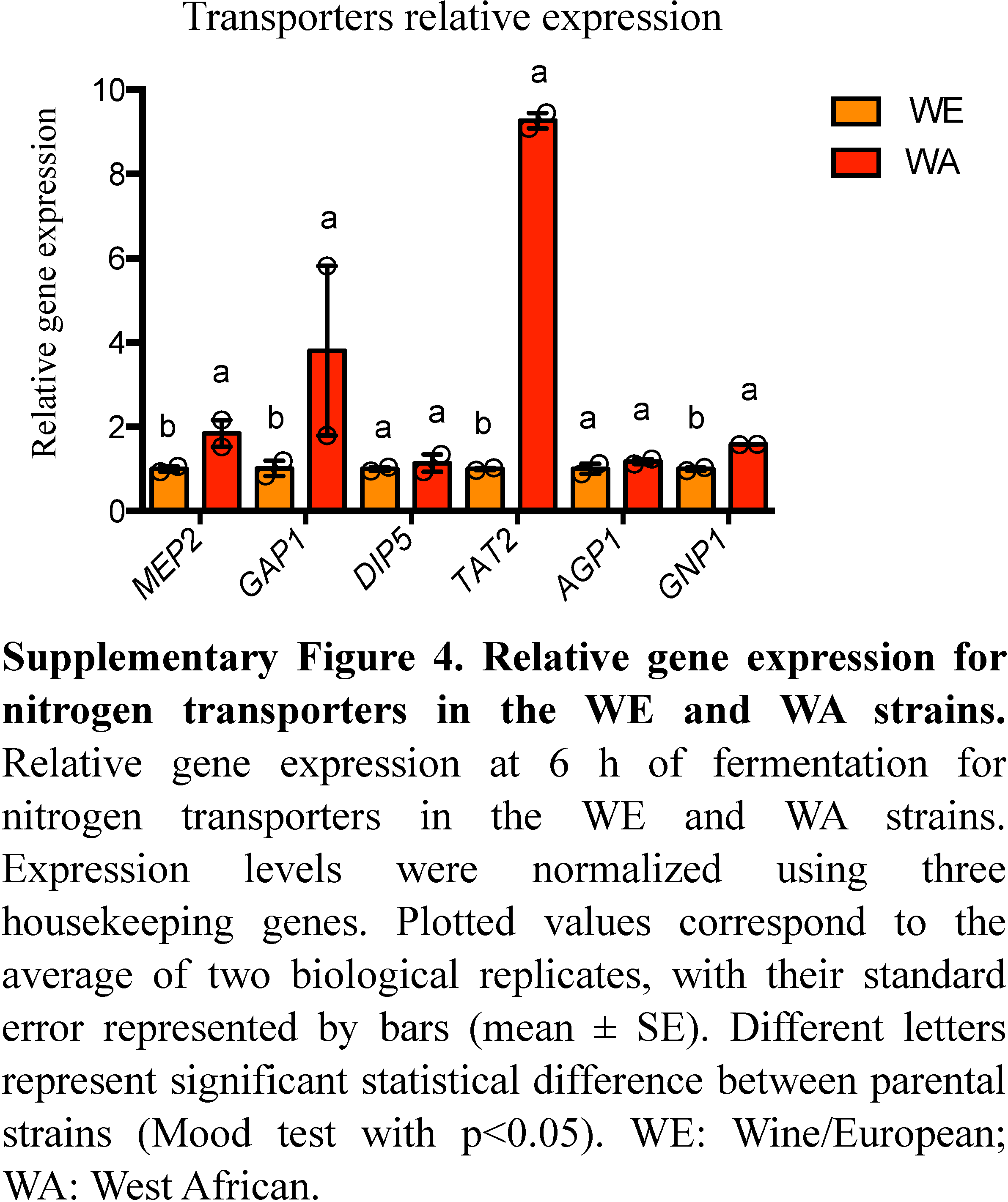

Supplement: Supplementary file 8 [file Image_4.TIF]

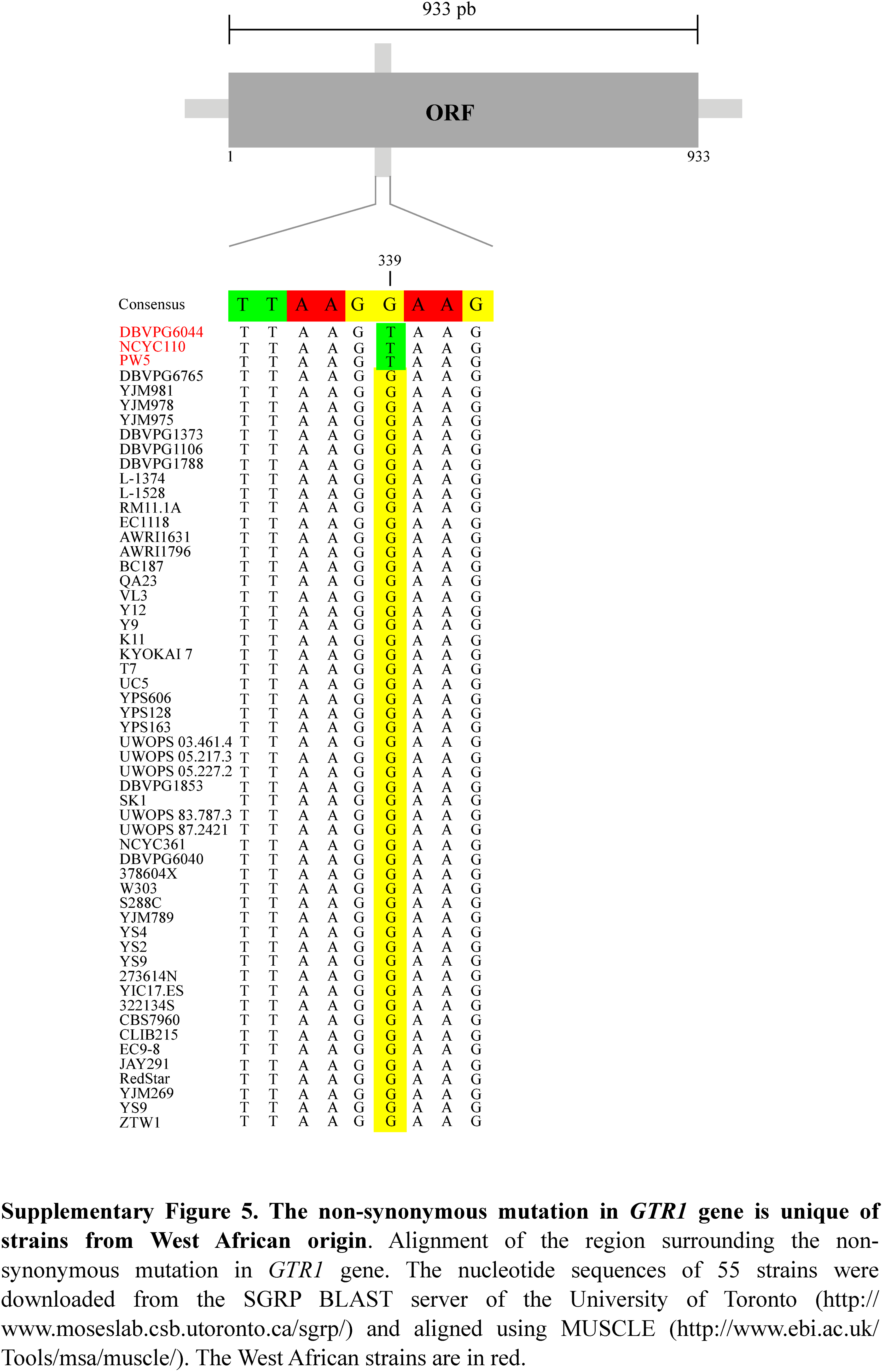

Supplement: Supplementary file 9 [file Image_5.TIF]
